# Modelling the ancestral sequence distribution and model frequencies in context-dependent models for primate non-coding sequences

**DOI:** 10.1186/1471-2148-10-244

**Published:** 2010-08-10

**Authors:** Guy Baele, Yves Van de Peer, Stijn Vansteelandt

**Affiliations:** 1 Department of Plant Systems Biology, VIB, B-9052 Ghent, Belgium; 2 Bioinformatics and Evolutionary Genomics, Department of Molecular Genetics, Ghent University, B-9052 Ghent, Belgium; 3 Department of Applied Mathematics and Computer Science, Ghent University, Krijgslaan 281 S9, B-9000 Ghent, Belgium

## Abstract

**Background:**

Recent approaches for context-dependent evolutionary modelling assume that the evolution of a given site depends upon its ancestor and that ancestor's immediate flanking sites. Because such dependency pattern cannot be imposed on the root sequence, we consider the use of different orders of Markov chains to model dependence at the ancestral root sequence. Root distributions which are coupled to the context-dependent model across the underlying phylogenetic tree are deemed more realistic than decoupled Markov chains models, as the evolutionary process is responsible for shaping the composition of the ancestral root sequence.

**Results:**

We find strong support, in terms of Bayes Factors, for using a second-order Markov chain at the ancestral root sequence along with a context-dependent model throughout the remainder of the phylogenetic tree in an ancestral repeats dataset, and for using a first-order Markov chain at the ancestral root sequence in a pseudogene dataset. Relaxing the assumption of a single context-independent set of independent model frequencies as presented in previous work, yields a further drastic increase in model fit. We show that the substitution rates associated with the CpG-methylation-deamination process can be modelled through context-dependent model frequencies and that their accuracy depends on the (order of the) Markov chain imposed at the ancestral root sequence. In addition, we provide evidence that this approach (which assumes that root distribution and evolutionary model are decoupled) outperforms an approach inspired by the work of Arndt et al., where the root distribution is coupled to the evolutionary model. We show that the continuous-time approximation of Hwang and Green has stronger support in terms of Bayes Factors, but the parameter estimates show minimal differences.

**Conclusions:**

We show that the combination of a dependency scheme at the ancestral root sequence and a context-dependent evolutionary model across the remainder of the tree allows for accurate estimation of the model's parameters. The different assumptions tested in this manuscript clearly show that designing accurate context-dependent models is a complex process, with many different assumptions that require validation. Further, these assumptions are shown to change across different datasets, making the search for an adequate model for a given dataset quite challenging.

## Background

Over the past decades, context-dependent evolutionary models have been the subject of an increasing number of studies. These studies demonstrate that the assumption of site-independent evolution is overly restrictive and that evolutionary models that take into account context-dependencies greatly improve model fit. Most context-dependent models have a temporal aspect in that the evolution of a given site over a branch is assumed to depend upon its immediate neighbours at the start of the branch. Specifically, context-dependent models are useful when studying mammalian genomes, due to the extensive methylation of C in CG doublets, which could make such Cs hotspots for mutation (see e.g. [1], for a review).

By allowing for mutations of both single nucleotides and pairs of neighbouring nucleotides, Arndt et al. [2] considered the evolution of an initial random sequence of nucleotides in discrete time steps, according to a set of update rules. Arndt et al. [2] further computed dinucleotide odds ratios to measure whether a given dinucleotide pair is over- or underrepresented and concluded that these correctly capture the strong underrepresentation of the CpG-dinucleotides in the human chromosome 21.

Hwang and Green [3] not only allow the evolution of a given site over a branch to depend upon its immediate neighbours at the start of the branch but further partition each branch into two or more small discrete time units, when the length of that branch is sufficiently long, in order to accurately approximate the continuous-time Markov substitution process. The authors estimate separate context-dependent rate matrices for five different clades and for a sixth group comprising the remaining ancestral branches. Hwang and Green [3] further model a dependency pattern along the sequence at the ancestral root using a second-order Markov chain.

Empirical studies (see e.g. [4-8]) have shown that the preceding or succeeding bases in a sequence have a large influence on the occurrence of a base, both in coding and non-coding sequences. This suggests the importance of modelling dependencies along the sequence. For example, Erickson and Altman [9] studied the nucleotide sequence of the RNA of the bacteriophage MS2 using Markov chains found that the total nucleotide sequence of the RNA of the bacteriophage MS2 showed very significant second-order dependence. Blaisdell [10] studied sixty-four eukaryotic nuclear DNA sequences, half of them coding and half non-coding using first-, second- and third-order Markov chains and found significant statistical evidence in favour of a third-order Markov chain in 5 of the 10 non-coding sequences longer than 1400 bases, suggesting that most sequences required at least second-order Markov chains for their representation while some required chains of third order.

In previous work [1], we have introduced a context-dependent model of evolution which allows the evolution of a site across a branch to depend upon the identities of its two immediate flanking bases at the start of the branch. Since the root of the tree does not have any ancestral sequence, we have assumed a site-independent distribution at the ancestral root sequence using a set of context-independent model frequencies, which is also used in the estimation of the context-dependent substitution rates. In this paper, we relax this assumption by modelling Markov chains of varying orders at the ancestral root sequence, thus modelling a context-dependency scheme across the entire tree. We also show that the assumption of using a single set of model frequencies for analyzing context-dependencies is overly restrictive. We allow for a set of context-dependent model frequencies which aims to accurately model the context-specific sequence composition throughout the underlying tree and test the performance of such a model by calculating the appropriate (log) Bayes Factors using thermodynamic integration [11]. We show that context-dependent model frequencies drastically increase the model fit and allow for accurate estimation of the parameters involved in specifying the ancestral root distribution. We assess the performance of an ancestral sequence composition that is coupled to the context-dependent model, inspired by the approach of Arndt et al. [2] and provide evidence that a decoupled root distribution outperforms a coupled root distribution. We compare the parameter estimates of our model for two approaches, i.e. a first approach that allows the evolution of a given site to depend only upon the identities of its immediate flanking bases at the start of a branch versus a second approach which partitions each branch into two or more parts to better accommodate evolving flanking bases across the branch.

## Methods

### Data

We have used a first dataset consisting of 10 vertebrate species (human, chimpanzee, gorilla, orang-utan, baboon, macaque, vervet, marmoset, dusky titi and squirrel monkey), as in Baele et al. [1]. In terms of the sequences used, this dataset is a subset of the alignment analyzed in the work of Hwang and Green [3] and Margulies et al. [12], containing sequences all orthologous to a ~1.9-Mb region on human chromosome 7q31.3 which contains the cystic fibrosis transmembrane conductance regulator gene (CFTR). We have extracted the ancestral repeats, transposons that appear to have been dispersed, and then become inactive, prior to the divergence of the species in question, and that are believed to have been evolving more or less neutrally since that time ([13]; see [1], for details on the preparation of the dataset). The resulting ancestral repeats dataset consists of the same type of sites as in the work of Siepel and Haussler [13], but contains only primate sequences. The dataset consists of 114,726 sites for each of the 10 sequences and is analyzed using the following rooted tree topology (((((human, chimpanzee), gorilla), orang-utan), ((baboon, macaque), vervet)), ((marmoset, dusky titi), squirrel monkey)).

A second dataset consists of the ψη-globin pseudogene sequences of six primates (human *[Homo Sapiens]*, chimpanzee *[Pan Troglodytes]*, gorilla *[Gorilla Gorilla]*, orang-utan *[Pongo Pygmaeus]*, rhesus monkey *[Macaca Mulatta] *and spider monkey *[Ateles Geoffroyi])*, containing 6,166 nucleotides in each sequence. We have used the fixed consensus tree shown in the work of Yang [14]: ((((human, chimpanzee), gorilla), orang-utan), (rhesus monkey, spider monkey)).

### Bayesian Markov Chain Monte Carlo using data augmentation

Bayesian inference of phylogeny is based on a quantity called the posterior probability function of a tree, in the same way as maximum-likelihood inference is based on the likelihood function. While the posterior probability is generally tedious to calculate, simulating from it is relatively easy through the use of Markov chain Monte Carlo (MCMC) methods ([15,16]). Relaxing the assumption of independent evolution leads to computational difficulties, which we handle via a data augmentation scheme [17]. Let *θ *be the collection of unknown parameters indexing the evolutionary model of interest, *Y_obs _*the observed nucleotide sequences (i.e., the observed data) and *Y_mis _*the unknown ancestral sequences (i.e., the missing data). The observed-data posterior  is intractable under our model because it involves the likelihood of the observed data which is computationally cumbersome. However, when *Y_obs _*is "augmented" by a random draw for *Y_mis _*from the distribution *f*(*Y*_*mis*_|*Y*_*obs*_, *θ*) of the ancestral sequences, the resulting complete-data posterior *f*(*θ*|*Y_obs,_Y_mis_*) becomes tractable. A detailed discussion of the data augmentation approach in our proposed Bayesian Markov chain Monte Carlo approach and in the thermodynamic integration framework for model comparison can be found in previous work [1,18].

### Context-dependent modelling assumptions

We provide a model-based approach to test the assumptions put forward in the empirical research of Blaisdell [10] and assume zero-, first-, second- and third-order Markov chains to specify the probability that a given base appears at a given site in the (ancestral) root sequence, given the identities of its preceding sites (i.e. the nucleotides occupying the preceding sites in the ancestral root sequence). For example, a second-order Markov chain is a temporal or spatial sequence of events characterized by the property that the outcome of any event in the chain may be dependent upon (e.g. influenced by) the two events immediately preceding it, but has no residual dependence on any further events preceding those. In other words, in the ancestral root sequence the probability that a given site occurs depends on the identities of the sites preceding it (i.e. which nucleotides precede the given site) in that same ancestral sequence. Arndt et al. [2] used a two-cluster approximation to calculate dinucleotide frequencies, yielding an exact solution if the stationary state of the mutation process is a first-order Markov chain, while Hwang and Green [3] modelled the distribution of bases at the ancestral root as a second-order Markov chain.

In the case of a zero-order Markov chain, one set of frequencies *π*_*ROOT *_= {*π*_*A*_, *π*_*C*_, *π*_*G*_, *π*_*T*_} is used to specify the distribution at the root sequence. When a first-order Markov chain is used, four independent sets of frequencies are required: *π*_*ROOT *_= {*π*_*X|A*_, *π*_*X|C*_, *π*_*X|G*_, *π*_*X|T*_}, with *X *∈ {*A*, *C*, *G*, *T*} the identity of the site preceding the given site. Likewise, a second-order *π*_*ROOT *_= {*π*_*YX|A*_, *π*_*YX|C*_, *π*_*YX|G*_, *π*_*YX|T*_} Markov chain and a third-order *π*_*ROOT *_= {*π*_*ZYX|A*_, *π*_*ZYX|C*_, *π*_*ZYX|G*_, *π*_*ZYX|T*_} Markov chain is specified with *Y *∈ {*A*, *C*, *G*, *T*} the identity of the site 2 positions prior to the given site and *Z *∈ {*A*, *C*, *G*, *T*} the identity of the site 3 positions prior to the given site. Zero-, first-, second- and third-order Markov chains increase the parameter space with respectively 1, 4, 16 and 64 sets of base frequencies (i.e., 4, 16, 64 and 256 parameters). Note that our notation for the distribution at the root sequence differs from the typical notation for conditional probabilities in that we put the conditional part before the 'pipe' symbol ('|').

To study the context-dependent substitution process across the remainder of the underlying tree, we estimate a general time-reversible model (GTR; 5 free evolutionary parameters and 3 free base frequency parameters; [19]) in each of the 16 possible neighbouring base combinations, yielding a context-dependent model consisting of 160 parameters (or 5 free evolutionary parameters and 3 free base frequency parameters to describe the GTR model in each context). In each context (i.e. in each of the 16 neighbouring base combinations), the general time-reversible model has the following substitution rates in the case of context-dependent model frequencies *π*_*XY *_= {*π*_*X*|*A*|*Y*_, *π*_*X*|*C*|*Y*_, *π*_*X*|*G*|*Y*_, *π*_*X*|*T*|*Y*_} and rate exchangeability parameters *q_X|*W*→*Z*|Y_*, with *X *(before the first 'pipe' symbol '|') the 5' and *Y *(after the second 'pipe' symbol '|') the 3' neighbouring base at the ancestral sequence of the branch (i.e. *X*, *Y *∈ {*A*, *C*, *G*, *T*}) and *W *the base at the start of a branch and *Z *the base at the end of that branch (the second 'pipe' symbol hence differs from the typical notation for conditional probabilities). These parameters are estimated for each context independently:.

Let *θ *be the collection of terms of the off-diagonal elements of the matrix above (with the starting base indicated by the row index and the resulting base indicated by the column index), each multiplied by the probability that one would start with that starting base (see e.g. [20]). This context-dependent model, although consisting of a collection of reversible models, allows for describing non-reversible evolutionary phenomena, such as the CpG-methylation-deamination process (see e.g. [21]). The context-dependent evolutionary model and the Markov chain(s) used to estimate the ancestral root distribution are hence decoupled (i.e. estimated independently from one another). Note that in the case of context-independent model frequencies, the model simplifies to the model in [1]. Throughout the remainder of this paper, we will use the notation *r_X|AC|Y_*, *r_X|AG|Y_*, *r_X|AT|Y_*, *r_X|CA|Y_*, *r_X|CG|Y_*, *r_X|CT|Y_*, *r_X|GA|Y_*, *r_X|GC|Y_*, *r_X|GT|Y_*, *r_X|*TA*|Y _*, *r_X|*TC*|Y _*and *r_X|*TG*|Y _*to indicate the substitution rates between two nucleotides. For instance, *r*_*X*|*AC*|*Y *_= *π*_*X*|*AC*|*Y *_. *q*_*X*|*A*→*C*|*Y *_denotes the substitution rate from A to C with *X *the 5' and *Y *the 3' neighbouring base at the ancestral sequence of the branch. When it is clear which neighbouring bases are meant or when no context-effects are assumed, we will use the notation *r_AC_*, *r_AG_*, *r_AT_*, *r_CA_*, *r_CG_*, *r_CT_*, *r_GA_*, *r_GC_*, *r_GT_*, *r_TA_*, *r_TC_*, and *r_TG _*to indicate the substitution rates.

Further, we assume a fixed underlying phylogenetic tree structure (see the Data section and [12]) for the evolutionary relationship between the species in our dataset. The branch lengths of the tree are estimated in our MCMC-run, along with all the other parameters of the context-dependent evolutionary model.

### Prior Distributions

Let *T *be the set of branch lengths with *t_b_*(*t_b _*≥ 0) one arbitrary branch length and *μ *a hyperparameter in the prior for *t_b _*in *T*. The following prior distributions *q*(.) are chosen for our analysis, with Γ(.) the Gamma function. Dirichlet priors (which are uninformative priors) assign densities to groups of parameters that measure proportions (i.e., parameters that must sum to 1). For each set of model frequencies of which the ancestral root sequence is composed, the following prior distribution is assumed:

For the model parameters of each context (i.e. neighbouring base combination) independently, the following prior distribution is assumed (see e.g. [22] and Additional file 1):

For each of the frequencies in each neighbouring base combination, with *X *the 5' and *Y *the 3' neighbouring base at the ancestral sequence of the branch (i.e. *X*, *Y *∈ {*A*, *C*, *G*, *T*}), the following prior distribution is assumed:

Further, branch lengths are assumed i.i.d. given *μ*:

*t_b_*|*μ *~Exponential (*μ*),  for each *t_b _*in *T*

and

*μ *~Inv-gamma (2.1, 1.1), , *μ *> 0.

### Bayes Factor Calculation

By allowing for context-dependent evolution, evolutionary models become more parameter-rich. As previously discussed [23], consistency problems may arise with such high-dimensional models, along with potential computational burdens. In view of this, a model-selection approach should be used that penalizes the addition of extra parameters unless there is a sufficiently impressive improvement in fit between model and data [23]. To compare the different assumptions concerning the root distribution, we have calculated (log) Bayes Factors [24]. Log Bayes Factors are typically divided into 4 categories depending on their value: from 0 to 1, indicating nothing worth reporting; from 1 to 3, indicating positive evidence of one model over the other; from 3 to 5, indicating strong evidence of one model over the other; and larger than 5, indicating very strong evidence of one model over the other [24]. We have chosen to calculate Bayes Factors using thermodynamic integration [11], since the traditional harmonic mean estimator of the marginal likelihood systematically favors parameter-rich models and is hence unfit to compare these complex context-dependent models. We have used the model-switch integration method and have performed bidirectional checks, i.e., we have calculated both annealing and melting integrations under various settings to obtain very similar runs, as suggested in the work of Rodrigue et al. [25]. When comparing different models, we report (log) Bayes Factor estimates for both annealing and melting integrations, as well as their mean. More details concerning the Bayes Factor calculation using a data augmentation approach can be found in Baele et al. [18].

### Coupled root distribution and context-dependent evolutionary model

Our approach for estimating context-dependent evolutionary patterns across a phylogenetic tree separates the estimation of the root distribution probabilities from the estimation of the context-dependent evolutionary patterns. This approach is similar to that of Hwang and Green [3] but clearly different from the approach of Arndt et al. [2] and Jensen and Pedersen [26]. Arndt et al. [2] derive the root distribution probabilities from the substitution process. Our substitution probabilities are different from theirs, as the dependencies cascade from the leaves of the tree up to its root, whereas the substitution probabilities in Arndt et al. [2] do not. In view of this, we integrated both approaches by means of a two-cluster approximation (see e.g. [27]) to derive the root distribution probabilities from our context-dependent evolutionary model. We focus here on deriving formulas for calculation of a first-order root distribution (with the 'pipe' symbol '|' denoting a traditional conditional probability) using the context-dependent model frequencies present in the context-dependent evolutionary model, with *a_i _*the identity of the base at a given site *i*, *p*(*a_i_*) the probability of observing *a_i_*, *p*(*a_i_*|*a*_*i*-1_) the probability of observing *a_i _*when the preceding base is *a*_*i*-1_, *p*(*a_i _*| *a*_*i*-1_, *a*_*i*+1_) the probability of observing *a_i _*when the preceding base is *a*_*i*-1 _and the succeeding base is *a*_*i*+1 _(i.e. the context-dependent model frequencies) and p(*a*_*i*+1 _| *a*_*i*-1 _) the probability of observing *a*_*i*+1 _when its second left-most base is *a*_*i*-1_:

with:.

This last equation can be rewritten using the two-cluster approximation as:

Substitution in the expression for *p*(*a_i_*|*a*_*i*-1_) then yields the expression for the first-order root distribution probabilities. Since analytic solutions were not possible, we used an iterative approach based on successive approximation (see e.g. [28]) to solve this system in each iteration of our MCMC approach. For each approximation we have used five iterations (in most cases, two were sufficient) to make the system converge towards a new solution.

### Simulation studies

We have performed two series of simulation experiments to examine the accuracy of our MCMC-approach and to assess whether our results might be influenced by the choice of prior distributions. The first series of simulations focuses on a second-order ancestral root distribution decoupled from the context-dependent evolutionary model while the second series of simulations focuses on a first-order ancestral root distribution coupled to the context-dependent model. Both simulations show that our method is able to reliably infer root distribution estimates, context-dependent model parameters and frequencies, as well as branch lengths from a large dataset. For more information on the set-up of the simulations, as well as the results of both simulations, we refer to Additional file 1, Additional file 2 and Additional file 3.

### Continuous-time approximation

In view of the computational complexity, we make the weak assumption that the identities of the immediate flanking neighbors remain constant across each branch of the tree. As the dataset analyzed in this paper contains closely related organisms, i.e. short internal and terminal branches will be estimated for the given tree topology, we expect that this assumption will not lead to drastically different parameter estimates compared to a branch-partitioning approach such as the one proposed by Hwang and Green [3]. Indeed, while this assumption makes our approach less biologically realistic, branch-partitioning is most likely only required for longer branches (i.e. branches longer than or equal to 0.005 using the approach of Hwang and Green [3,20]). To assess the need for partitioning the branches in our dataset, we split each branch into two or more parts of equal length such that the average substitution rate per time unit is smaller than or equal to 0.005 [3] and compare all the parameter estimates of our context-dependent model with a second-order Markov chain at the ancestral root to our approach without branch partitioning. Instead of estimating context-dependent substitution patterns on 18 branches, these patterns now have to be estimated on 52 branch parts, which leads to an almost 3-fold increase in computation time. The branch-splitting approach of Hwang and Green is a discrete-time method. For a comparison of both continuous and discrete-time methods to sample substitution histories along branches, in terms of performance and accuracy, we refer to the work of de Koning et al. [29]. Apart from comparing the parameter estimates, we have also calculated an additional (log) Bayes Factor of our context-dependent model with a second-order Markov chain against an independent GTR model, with the branch lengths partitioned.

## Results

### Ancestral Repeats

#### Independent model frequencies: Bayes Factors

**Figure 1:**
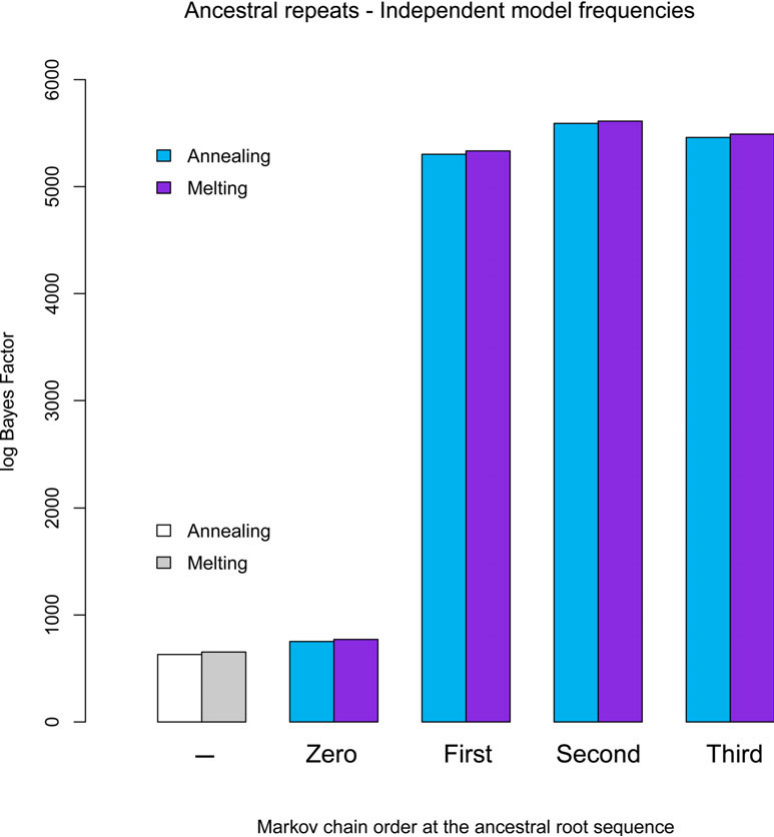
**Ancestral repeats: Influence of root sequence distributions on model fit (against independence throughout the entire tree) using independent model frequencies**. Various orders of Markov chains for the ancestral root sequence are tested against the assumption of site-independent evolution throughout the entire tree, revealing that a second-order Markov chain yields the largest increase in model fit vis-à-vis the independent GTR model. The first model comparison (of which the order is indicated by '-') does not assume a separate Markov chain at the ancestral root but uses the independent model frequencies to describe the ancestral root sequence. Both annealing and melting schemes are shown for each model comparison. The 95% confidence intervals are at most 30 log units wide (not shown).

In Figure 1, we calculate the increase in model fit vis-à-vis the general time-reversible model, brought about by relaxing the assumption of site-independent evolution at the ancestral root sequence (the numerical results are reported in Table S1 in Additional file 1). The assumption of a separate set of base frequencies at the root already provides a significant increase in terms of model fit over the model of Baele et al. [1], even though such a zero-order Markov chain still assumes a site-independent distribution at the ancestral root sequence. The first- and second-order Markov chain at the root sequence yield a phenomenal increase in terms of model fit. The assumption of a third-order Markov chain at the root sequence yields no further improvement, which may be related to the corresponding drastic increase in number of parameters.

#### Context-dependent model frequencies: Bayes Factors

**Figure 2:**
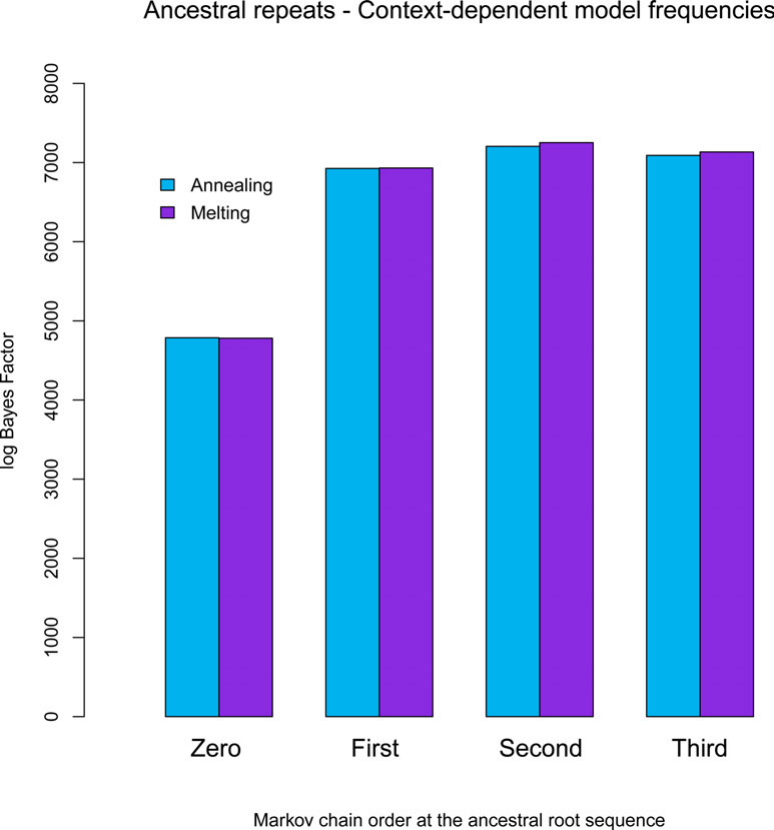
**Ancestral repeats: Influence of root sequence distributions on model fit (against independence throughout the entire tree) using context-dependent model frequencies**. Various orders of Markov chains for the ancestral root sequence are tested against the assumption of site-independent evolution throughout the entire tree using the context-dependent model with context-dependent model frequencies, revealing once again that a second-order Markov chain yields the largest increase in model fit vis-à-vis the independent GTR model. Both annealing and melting schemes are shown for each model comparison. The 95% confidence intervals are at most 30 log units wide (not shown).

We have calculated (log) Bayes Factors for our context-dependent model with context-dependent model frequencies and Markov chains of different orders at the ancestral root sequence in the same way as reported above, with the general time-reversible model as the reference model. The results are shown in Figure 2 (the numerical results are reported in Table S2 in Additional file 1). Even when assuming a site independent distribution at the ancestral root sequence, a drastic increase in model fit is realized through context-dependent model frequencies. As when assuming independent model frequencies, optimal fit is reached when assuming a second-order Markov chain at the ancestral root sequence, although the increase in model fit is now higher than when assuming independent model frequencies.

#### Coupled root distribution and context-dependent evolutionary model: Bayes Factor

Inspired by the work of Arndt et al. [2], we have calculated the log Bayes Factor for this first-order successive approximation approach with coupled root distribution against the independent GTR model, resulting in an annealing estimate of 6648.61 ([6628.69; 6668.52]) and a melting estimate of 6682.52 ([6659.95; 6705.09]), yielding a bidirectional mean log Bayes Factor of 6665.56 log units. We conclude that our approach to couple the root distribution to the evolutionary model, inspired by the work of Arndt et al. [2], yields a suboptimal model fit for this data.

#### Continuous-time approximation: Bayes Factor

As mentioned above, the context-dependent model with context-dependent model frequencies and a second-order Markov chain at the ancestral root sequence yields a a bidirectional mean log Bayes Factor of 7221.47 log units, compared to the independent GTR model (7218.01 ([7200.34; 7235.68]) log units for the annealing scheme and 7224.94 ([7207.16; 7242.71]) log units for the melting scheme). We have recalculated the log Bayes Factor for this model, this time splitting the branches where appropriate. Due to the use of a full data likelihood (as a consequence of using a data augmentation approach), the branches under both the context-dependent and independent model being compared need to be split into parts as otherwise the two likelihoods corresponding to the two models would be incomparable. The independent model will however not benefit from the branch partitioning, as it is an approach aimed at approximating the continuous distribution of the context-dependent Markov substitution process. The split branches model comparison yields a bidirectional mean log Bayes Factor of 7626.70 log units, compared to the independent GTR model (7617.66 ([7600.11; 7635.21]) log units for the annealing scheme and 7635.74 ([7617.94; 7653.55]) log units for the melting scheme). This difference of approximately 405 log units cannot solely be attributed to the difference in evolutionary model estimates, given that these differ only slightly between both approaches. The main reason for this significant difference is hence likely the more accurate approximation of the context-dependent Markov substitution process by allowing the ancestral sequences to change in between the internal speciation nodes (which is also responsible for the small differences in model estimates).

#### Independent model frequencies: root distribution estimates

From previous work [1], we have obtained the stationary distribution given (with accompanying 95% credibility intervals) by: *π*_*A *_= 0.300 ([0.297; 0.302]), *π_C _*= 0.192 ([0.190; 0.194]), *π_G _*= 0.192 ([0.190; 0.194]) and *π_T _*= 0.317 ([0.314; 0.320]). These estimates, as well as those presented in the remainder of this work, were obtained by performing 100,000 MCMC iterations, discarding the first 20,000 as the burn-in sequence and calculating the mean of the remaining 80,000 samples.

Modelling a zero-order Markov chain along the ancestral root sequence still assumes a site-independent distribution but relaxes the stationarity assumption by allowing for a different set of base frequencies to estimate the evolutionary models that describe substitutions in the remainder of the tree. By comparing the two sets of base frequencies, one can assess the restrictions imposed by assuming a stationary distribution across the entire tree. The estimates for the base frequencies along the ancestral root sequence (with accompanying 95% credibility intervals) *π_A _*= 0.295 ([0.293; 0.298]), *π_C _*= 0.198 ([0.195; 0.200]), *π_G _*= 0.196 ([0.194; 0.199]), *π_T _*= 0.311 ([0.3087; 0.314]) are different from those used throughout the remainder of the tree *π_A _*= 0.317 ([0.307; 0.328]), *π_C _*= 0.161 ([0.155; 0.168]), *π_G _*= 0.167 ([0.161; 0.174]), *π_T _*= 0.354 ([0.344; 0.365]).

Allowing for a first-order Markov chain along the sequence yields the four sets of base frequencies for the ancestral root sequence in Table 1. The estimates from the first-order Markov chain at the ancestral root sequence clearly show an important effect of mammalian evolution, i.e. the presence of a so-called CpG-effect (see e.g. [21]). Indeed, the probability of encountering a G at the ancestral root sequence when it is preceded by a C is extremely low (0.69%), which leads to increased probabilities of observing either A, C or T at the root sequence when the preceding site is a C. This confirms the result of Arndt et al. [2], who calculated dinucleotide frequencies and odds ratios and showed that the CpG dinucleotide is underrepresented in mammalian sequences as a result of the CpG-methylation-deamination process, although the estimate in Table 1 is much lower than theirs. This underscores the importance of (at least) a first-order Markov chain when modelling context-dependent evolution. The comparison of the remaining rows in Table 1 illustrates the presence of other evolutionary patterns. For example, while the probability of observing a C or a G in the ancestral root sequence is quite similar regardless of whether the preceding site is G or T, there is a higher probability of observing an A when the preceding site is G than when it is T.

**Table 1:** Ancestral repeats - First-order root sequence distribution

	Root			
***X***	***π***_***X|A***_	***π***_***X|C***_	***π***_***X|G***_	***π***_***X|T***_

A	0.3114 [0.3063; 0.3162]	0.1682 [0.1641; 0.1723]	0.2355 [0.2307; 0.2400]	0.2849 [0.2799; 0.2899]
C	0.3760 [0.3697; 0.3826]	0.2439 [0.2380; 0.2496]	0.0069 [0.0057; 0.0083]	0.3733 [0.3668; 0.3799]
G	0.3014 [0.2954; 0.3077]	0.1926 [0.1873; 0.1981]	0.2448 [0.2389; 0.2504]	0.2612 [0.2554; 0.2672]
T	0.2287 [0.2241; 0.2331]	0.1966 [0.1924; 0.2012]	0.2451 [0.2405; 0.2497]	0.3296 [0.3247; 0.3347]

	**Rest of the tree**			

	***π***_***A***_	***π***_***C***_	***π***_***G***_	***π***_***T***_

	0.3114 [0.3015; 0.3217]	0.1676 [0.1610; 0.1739]	0.1730 [0.1665; 0.1798]	0.3480 [0.3374; 0.3582]

Estimates (mean and accompanying 95% credibility interval) for the four sets of base frequencies at the ancestral root sequence and the set of base frequencies used in the remainder of the tree, for the context-dependent model with independent model frequencies and a first-order Markov chain at the ancestral root. The extremely low estimate for the probability of observing a G when the preceding base is a C, is a direct consequence of the CpG-methylation-deamination process in mammalian sequences. Note that the base frequencies used throughout the remainder of the tree differ from those when assuming a zero order Markov chain at the ancestral root sequence.

To assess whether the probability of observing a given nucleotide at a given site in the ancestral root sequence depends only on its immediate preceding site, we have also modelled a second-order Markov chain at the ancestral root sequence. This was also used by Hwang and Green [3], but without testing whether it was supported by the data. The estimates for the sixteen sets of base frequencies for the ancestral root sequence are shown in Table 2.

**Table 2:** Ancestral repeats - Second-order root sequence distribution

	Root			
***YX***	***π***_***YX|A***_	***π***_***YX|C***_	***π***_***YX|G***_	***π***_***YX|T***_

AA	0.3496 [0.3404; 0.3587]	0.1518 [0.1449; 0.1588]	0.2160 [0.2080; 0.2241]	0.2826 [0.2737; 0.2916]
CA	0.2437 [0.2343; 0.2533]	0.1987 [0.1899; 0.2074]	0.2648 [0.2554; 0.2748]	0.2928 [0.2831; 0.3028]
GA	0.3326 [0.3212; 0.3442]	0.1553 [0.1461; 0.1647]	0.2753 [0.2644; 0.2864]	0.2367 [0.2261; 0.2473]
TA	0.3148 [0.3044; 0.3253]	0.1679 [0.1594; 0.1766]	0.1983 [0.1894; 0.2075]	0.3190 [0.3083; 0.3298]

AC	0.4062 [0.3929; 0.4199]	0.2295 [0.2177; 0.2406]	0.0092 [0.0064; 0.0124]	0.3551 [0.3422; 0.3681]
CC	0.3791 [0.3661; 0.3922]	0.2357 [0.2242; 0.2473]	0.0057 [0.0036; 0.0083]	0.3795 [0.3665; 0.3927]
GC	0.3763 [0.3614; 0.3916]	0.2541 [0.2407; 0.2679]	0.0040 [0.0020; 0.0065]	0.3656 [0.3503; 0.3803]
TC	0.3487 [0.3373; 0.3603]	0.2554 [0.2448; 0.2664]	0.0083 [0.0060; 0.0109]	0.3875 [0.3757; 0.3994]

AG	0.3153 [0.3051; 0.3260]	0.1941 [0.1853; 0.2032]	0.2464 [0.2368; 0.2562]	0.2441 [0.2343; 0.2537]
CG	0.2557 [0.1796; 0.3398]	0.1960 [0.1272; 0.2723]	0.2617 [0.1887; 0.3433]	0.2866 [0.2044; 0.3719]
GG	0.3081 [0.2955; 0.3212]	0.2049 [0.1939; 0.2163]	0.2400 [0.2281; 0.2517]	0.2471 [0.2353; 0.2592]
TG	0.2844 [0.2747; 0.2944]	0.1839 [0.1754; 0.1926]	0.2462 [0.2368; 0.2556]	0.2855 [0.2757; 0.2953]

AT	0.2575 [0.2484; 0.2668]	0.1657 [0.1582; 0.1735]	0.2495 [0.2405; 0.2586]	0.3272 [0.3175; 0.3368]
CT	0.1968 [0.1879; 0.2059]	0.2200 [0.2111; 0.2290]	0.2630 [0.2533; 0.2725]	0.3202 [0.3100; 0.3307]
GT	0.2322 [0.2208; 0.2437]	0.1919 [0.1812; 0.2025]	0.2814 [0.2694; 0.2938]	0.2945 [0.2824; 0.3069]
TT	0.2256 [0.2179; 0.2334]	0.2077 [0.2000; 0.2153]	0.2110 [0.2036; 0.2187]	0.3558 [0.3472; 0.3646]

	**Rest of the tree**			

	***π_A _***	***π_C _***	***π _G _***	***π _T _***

	0.3114 [0.3016; 0.3211]	0.1681 [0.1617; 0.1746]	0.1721 [0.1658; 0.1785]	0.3484 [0.3385; 0.3589]

Estimates (mean and accompanying 95% credibility interval) for the sixteen sets of base frequencies at the ancestral root sequence and the set of base frequencies used in the remainder of the tree, for the context-dependent model with independent model frequencies and a second-order Markov chain at the ancestral root. The probabilities are grouped by the identity of the immediate preceding site. Note that the base frequencies used throughout the remainder of the tree are similar to those when assuming a first order Markov chain at the ancestral root sequence.

The estimates from the second-order Markov chain at the ancestral root sequence show additional differences in base frequencies when compared to the estimates from the first-order Markov chain. For example, there are large relative differences in the probabilities of observing a G when the preceding site is a C. When that C is preceded by an A, the probability of observing a G is more than twice as much as when C is preceded by G. Likewise, from Table 2 we see that the probability of observing an A equals 31.14% when its preceding site is an A. However, the site two positions away causes variation in this estimate, i.e. from 24.37% up to 34.96%. This illustrates the importance of using a second-order Markov chain at the ancestral root sequence.

#### Independent model frequencies: parameter estimates

**Figure 3:**
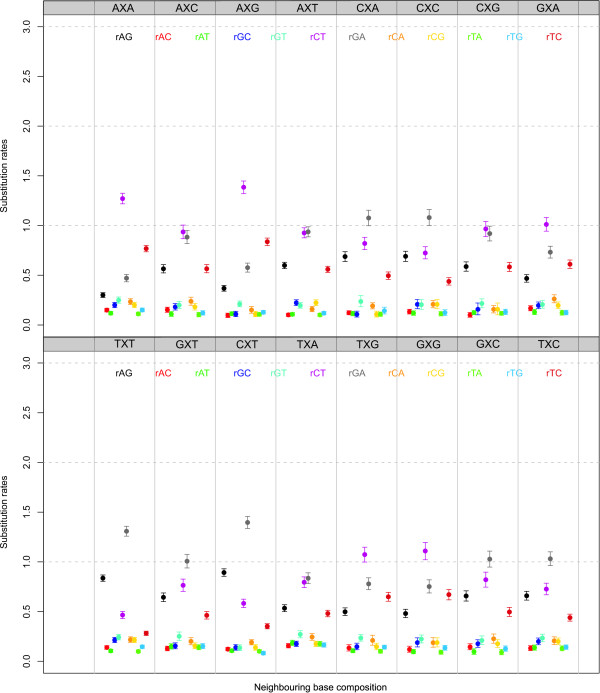
**Ancestral repeats: Substitution parameters for independent model frequencies and a second-order Markov chain at the root**. Substitution parameter estimates (mean and 95% credibility intervals) for each of the twelve entries of the general time-reversible model in each of the sixteen neighbouring base combination using independent model frequencies and a second-order Markov chain to specify the ancestral root distribution.

We have shown that the addition of a second-order Markov chain at the ancestral root sequence increases model fit drastically over a site-independent distribution at the root. To determine whether the inferred context-dependent parameters are influenced by modelling a second-order Markov chain at the ancestral root sequence we have performed two separate MCMC runs of 100,000 iterations, one for the independent general time-reversible model and one for the context-dependent model (using independent model frequencies) with a second-order Markov chain at the root. After discarding the first 20,000 iterations as the burn-in sequence, we have constructed posterior difference densities for each of the 192 entries in the context-dependent matrices. The resulting posteriors were used to determine a Bayesian p-value (see e.g. [30]) for each of the 192 entries to test whether the parameter estimates were significantly different between both MCMC runs. This approach resulted in the detection of 24 significantly differing evolutionary parameters (at a 5% level). Interestingly, all of these were found to include one of the transition parameters. Indeed, 4 out of 16 neighbouring base combinations showed different estimates for *r_AG _*(specifically: *r*_*A*|*AG*|*T*_, *r*_*C*|*AG*|*A*_, *r*_*C*|*AG*|*C *_and *r*_*C*|*AG*|*T*_), 6 out of 16 neighbouring base combinations showed different estimates for *r_TC _*(specifically: *r*_*A*|*TC*|*A*_, *r*_*A*|*TC*|*G*_, *r*_*A*|*TC*|*T*_, *r*_*C*|*TC*|*T*_, *r*_*G*|*TC*|*A *_and *r*_*T*|*TC*|*A*_) and 13 out of 16 neighbouring base combinations showed different estimates for *r_CT _*(*r*_*C*|*CT*|*A*_, *r*_*C*|*CT*|*C*_, and *r*_*G*|*CT*|*C *_did not). As the assumption of a second-order Markov chain at the ancestral root sequence results in a drastic increase in model fit over assuming a site independent distribution at that root sequence, we show the substitution rates for the 192 off-diagonal elements in our context-dependent model using independent model frequencies in Figure 3. The difference with the substitution rates shown in our previous work [1] is that Figure 3 shows the matrix entries of the evolutionary models (thus evolutionary parameters after being multiplied by the model frequencies).

#### Context-dependent model frequencies: root distribution estimates

As a second-order Markov chain at the ancestral root sequence yields the largest increase in model fit, we have re-estimated the corresponding root distribution probabilities when assuming context-dependent model frequencies. The results can be seen in Table 3. We compare them with the results reported in Table 2, which were obtained using a single set of independent model frequencies. Large relative differences occur in those combinations of sites involved in the CpG-methylation-deamination process. Indeed, the root distribution probabilities differ in the following combinations of preceding sites: AC, CC, GC, TC and CG. As each row in Table 3 sums to one, a difference in one probability estimate influences the three remaining probability estimates given the same combination of preceding sites.

**Table 3:** Ancestral repeats - Second-order root distribution estimates

*YX*	***π***_***YX|A***_	***π***_***YX|C***_	***π***_***YX|G***_	***π***_***YX|T***_
AA	0.3492 [0.3398; 0.3586]	0.1539 [0.1466; 0.1613]	0.2132 [0.2050; 0.2215]	0.2837 [0.2747; 0.2928]
CA	0.2453 [0.2355; 0.2552]	0.2036 [0.1942; 0.2133]	0.2639 [0.2536; 0.2742]	0.2872 [0.2767; 0.2981]
GA	0.3308 [0.3192; 0.3426]	0.1588 [0.1495; 0.1682]	0.2737 [0.2626; 0.2848]	0.2367 [0.2261; 0.2473]
TA	0.3134 [0.3031; 0.3237]	0.1698 [0.1613; 0.1788]	0.1977 [0.1888; 0.2068]	0.3191 [0.3083; 0.3298]

AC	0.3824 [0.3684; 0.3960]	0.2331 [0.2215; 0.2451]	0.0462 [0.0380; 0.0551]	0.3384 [0.3252; 0.3516]
CC	0.3579 [0.3447; 0.3715]	0.2379 [0.2259; 0.2497]	0.0406 [0.0331; 0.0486]	0.3636 [0.3501; 0.3772]
GC	0.3597 [0.3443; 0.3752]	0.2585 [0.2448; 0.2724]	0.0291 [0.0218; 0.0372]	0.3527 [0.3375; 0.3678]
TC	0.3344 [0.3228; 0.3458]	0.2587 [0.2480; 0.2695]	0.0290 [0.0236; 0.0347]	0.3779 [0.3661; 0.3896]

AG	0.3160 [0.3052; 0.3265]	0.1941 [0.1851; 0.2036]	0.2492 [0.2394; 0.2594]	0.2407 [0.2309; 0.2506]
CG	0.2122 [0.1726; 0.2542]	0.1598 [0.1237; 0.1990]	0.2467 [0.2025; 0.2934]	0.3813 [0.3310; 0.4327]
GG	0.3053 [0.2927; 0.3181]	0.2070 [0.1959; 0.2185]	0.2429 [0.2312; 0.2546]	0.2448 [0.2331; 0.2569]
TG	0.2842 [0.2741; 0.2945]	0.1857 [0.1771; 0.1946]	0.2499 [0.2402; 0.2598]	0.2802 [0.2699; 0.2904]

AT	0.2643 [0.2550; 0.2736]	0.1683 [0.1606; 0.1762]	0.2378 [0.2285; 0.2471]	0.3296 [0.3198; 0.3394]
CT	0.2025 [0.1934; 0.2117]	0.2247 [0.2152; 0.2342]	0.2514 [0.2416; 0.2614]	0.3214 [0.3112; 0.3320]
GT	0.2387 [0.2271; 0.2501]	0.1944 [0.1837; 0.2048]	0.2727 [0.2608; 0.2846]	0.2942 [0.2823; 0.3067]
TT	0.2295 [0.2216; 0.2372]	0.2106 [0.2030; 0.2183]	0.2051 [0.1974; 0.2126]	0.3548 [0.3460; 0.3637]

Estimates (mean and accompanying 95% credibility interval) for the sixteen sets of base frequencies at the ancestral root sequence under the context-dependent model with context-dependent model frequencies. The probabilities are grouped by the identity of the immediately preceding site.

Each of the four preceding site combinations AC, CC, GC, TC has a drastically different estimate for the probability of a G occurring at the succeeding site. This is in accordance with Arndt et al. [2], who considered a more approximate development based on dinucleotide frequencies and odds ratios conditional on the left preceding base (rather than the two left preceding bases). The estimates indicate a dependence of the probability of observing a G on the second left-most base. Indeed, the probability of observing a G at a given site is respectively 4.62% and 4.06% when AC and CC precede that site, but only 2.91% and 2.90% respectively, when GC and TC precede that site.

The decreased probabilities of observing a G at a given site when a C precedes it, is caused by the hypermutability of CpG in human and other species, which, in turn, is due to the fact that cytosine is methylated only in CpG dinucleotides (in vertebrates). Both cytosine and 5-methylcytosine undergo high rates of spontaneous hydrolytic deamination, but deamination of 5-methylcytosine produces thymine, and mismatch repair of *C *→ *T *transitions is less efficient than that of *C *→ *U *transitions (for more information, see [31]). Further, when CG precedes a given site, the probability of observing a C at that site drops from 19.60% when assuming a single set of independent model frequencies to 15.98% when assuming context-dependent model frequencies. These differences demonstrate the importance of relaxing the assumption of independent model frequencies.

#### Context-dependent model frequencies: parameter estimates

**Figure 4:**
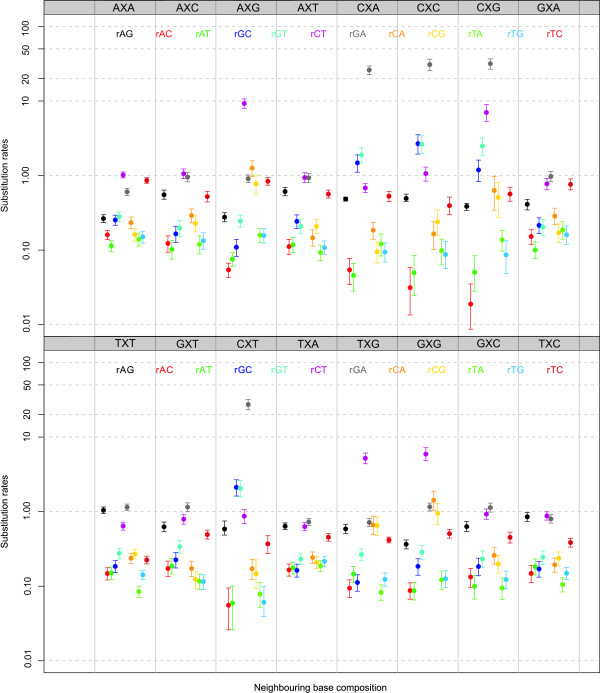
**Ancestral repeats: Substitution parameters for context-dependent model frequencies and a zero-order Markov chain at the root**. Substitution parameter estimates (mean and 95% credibility intervals) for each of the twelve entries of the general time-reversible model in each of the sixteen neighbouring base combination using context-dependent model frequencies and a zero-order Markov chain to specify the ancestral root distribution. Note that the log-scale is used for the y-axis.

Based on the estimated 192 off-diagonal matrix entries of our context-dependent model assuming context-dependent model frequencies, we constructed Bayesian p-values (see e.g. [30]) to test whether the parameters were significantly different between an MCMC assuming independence at the ancestral root (i.e., a zero-order Markov chain) and a second-order Markov chain. For matters of comparison, Figure 4 shows all the substitution rates of our context-dependent model using context-dependent model frequencies, assuming a zero-order Markov chain to describe the sequence composition of the ancestral root. It shows large variation in substitution rates across different neighbouring base combinations. Given the presence of the CpG-methylation-deamination process in mammals, we focus on the substitution rates from C to T (*r_CT_*) in the AXG, CXG, GXG and TXG contexts as well as their compensating substitution rates from G to A (*r_GA_*) in the respective CXT, CXG, CXC and CXA contexts. The mean substitution rates *r_CT _*are 9.26, 7.01, 5.91 and 5.19 for the AXG, CXG, GXG and TXG contexts, respectively, and 27.32, 31.62, 30.77 and 26.04 for the compensating substitution rates *r_GA _*in the CXT, CXG, CXC and CXA contexts, respectively. While the compensating substitution rates are clearly elevated, they can hardly be called compensating (rather over-compensating).

When comparing these estimates to the estimates of the matrix entries when assuming a second-order Markov chain at the ancestral root sequence, 27 significantly differing substitution rates are observed out of the 192 tested at the 5% significance level. Specifically, the following substitution rates were found to differ most (in terms of their p-values): *r_GA _*in contexts CXA, CXC, CXG and CXT; *r_CT _*in contexts CXA, AXG, CXG, GXG and TXG; *r_AT _*in contexts CXA, CXC, CXG and CXT; *r_AG _*in contexts CXC and CXT; *r_CA _*in context CXG; *r_TA _*in context CXG; *r_GT _*in context CXA; and *r_AC_*, *r_GC _*in contexts CXA, CXC, CXG and CXT and *r_CT _*in context CXG. All these differences are observed in those contexts related to the CpG-methylation-deamination process, as the increase in *r_CT _*and *r_GA _*substitution rates influences the remaining parameters of the evolutionary model.

**Figure 5:**
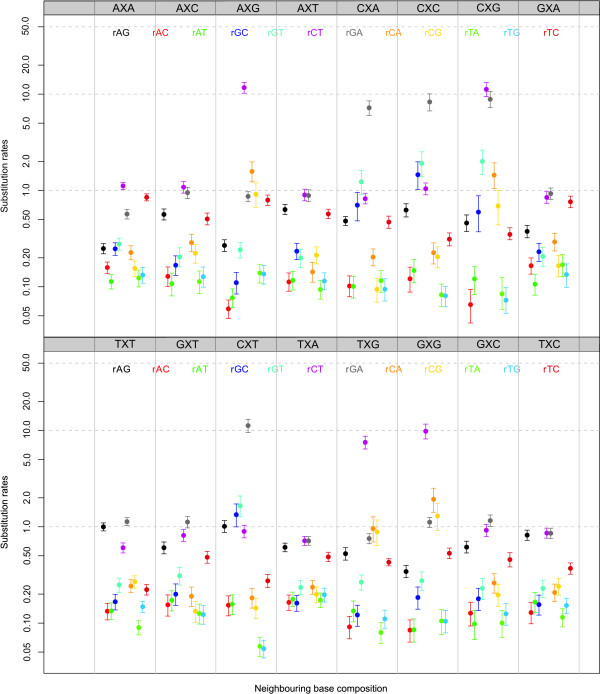
**Ancestral repeats: Substitution parameters for context-dependent model frequencies and a second-order Markov chain at the root**. Substitution parameter estimates (mean and 95% credibility intervals) for each of the twelve entries of the general time-reversible model in each of the sixteen neighbouring base combination using context-dependent model frequencies and a second-order Markov chain to specify the ancestral root distribution. Note that the log-scale is used for the y-axis.

However, as can be seen from Figure 2, a second-order Markov chain at the ancestral root sequence along with context-dependent model frequencies offers the largest increase in model fit when compared to the independent general time-reversible model. We show the substitution rates for the 192 off-diagonal elements in our context-dependent model using context-dependent model frequencies and a second-order Markov chain at the ancestral root sequence in Figure 5. The difference with employing a zero-order Markov chain at the ancestral root is staggering, specifically in terms of the substitution parameters that describe the CpG-effect. The mean substitution rates *r_CT _*are now 11.69, 11.22, 9.82 and 7.53 for the AXG, CXG, GXG and TXG contexts, respectively, versus 11.23, 8.84, 8.30 and 7.21 for the compensating substitution rates *r_GA _*in the CXT, CXG, CXC and CXA contexts, respectively.

The context-dependent substitution rates *r_CT _*clearly illustrate the hypermutability of CpG in mammals, as the *r_CT _*rates in the CpG-related contexts are about 10 times higher than those in other contexts. Further, they are in accordance with the root distribution estimates reported in the previous section. Indeed, the higher the context-dependent *r_CT _*rate, the lower the corresponding ancestral root distribution estimate as the mean substitution rates *r_CT _*of 11.69, 11.22, 9.82 and 7.53 for the AXG, CXG, GXG and TXG contexts correspond to the following probabilities of observing a G at a given site of 4.62%, 4.06%, 2.91% and 2.90%, when preceded by AC, CC, GC and TC respectively.

These results are clearly more indicative of the effect of compensating mutations at the opposite side of a stem region and are supported by the largest increase in model fit. However, as discussed by Green et al. [32] and Siepel and Haussler [13], three of the nine studied genes are located on the opposite strand from the other six so our data contains a mixture of bases that correspond to the transcribed and non-transcribed strands. This supports our findings of compensating substitution rates at opposing stem regions, although we find that the *r_CT _*substitution rate is between 4.1% and 26.9% higher than the compensating *r_GA _*substitution rate when a given site has a G as its 3' neighbour.

Finally, in Table S3 (in Additional file 1), we report the estimates for the context-dependent model frequencies for our optimal model. Note that these probabilities are conditional on the identities of the two immediate flanking bases, while those at the ancestral root sequence are dependent upon the two left flanking bases. The table reveals two sets of frequency estimates related to the CpG-methylation-deamination process: *π*_*A*|*C*|*G*_, *π*_*C*|*C*|*G*_, *π*_*G*|*C*|*G*_, *π*_*T*|*C*|*G *_and *π*_*C*|*G*|*A*_, *π*_*C*|*G*|*C*_, *π*_*C*|*G*|*G*_, *π*_*C*|*G*|*T*_. The estimates of *π*_*A*|*C*|*G *_and *π*_*C*|*G*|*T *_are nearly identical, which can be attributed to the fact that these frequencies represent compositional aspects of opposing sides of a stem region. This also holds for *π*_*T*|*C*|*G *_and *π*_*C*|*G*|*A *_but not for the other two pairs. The reason for *π*_*C*|*C*|*G *_and *π*_*C*|*G*|*G *_as well as *π*_*G*|*C*|*G *_and *π*_*C*|*G*|*C *_to differ is reflected in the estimated substitution rates in Figure 5. Indeed, for the CXG and GXG neighbouring base combinations there is a discrepancy between the *r*_*CT *_and *r*_*GA *_substitution rates corresponding to the difference in estimates for the context-dependent model frequencies.

#### Third-order Markov chains

While Bayes Factor calculations did not support the assumption of a third-order Markov chain at the ancestral root, this may be partly related to the inflation in number of parameters in such models. To gather additional information on the usefulness of a third-order Markov chain at the ancestral root sequence, we have therefore applied a four-dimensional (i.e. clustering *π_A_*, *π_C_*, *π_G_*, *π_T _*together) principal component clustering for the identity of the left-most base on which a given site is assumed to depend in a third-order Markov chain. We found that the most plausible combinations where the identity of the left-most nucleotide has no additional influence are: X-A-C, X-C-A, X-C-T, X-T-A and X-T-G (see Additional file 4). In other words, reducing the third-order Markov chain for these combinations to a second-order Markov chain may improve model fit. We provide additional information on this approach in Additional file 1.

#### Coupled root distribution and context-dependent evolutionary model: parameter estimates

**Figure 6:**
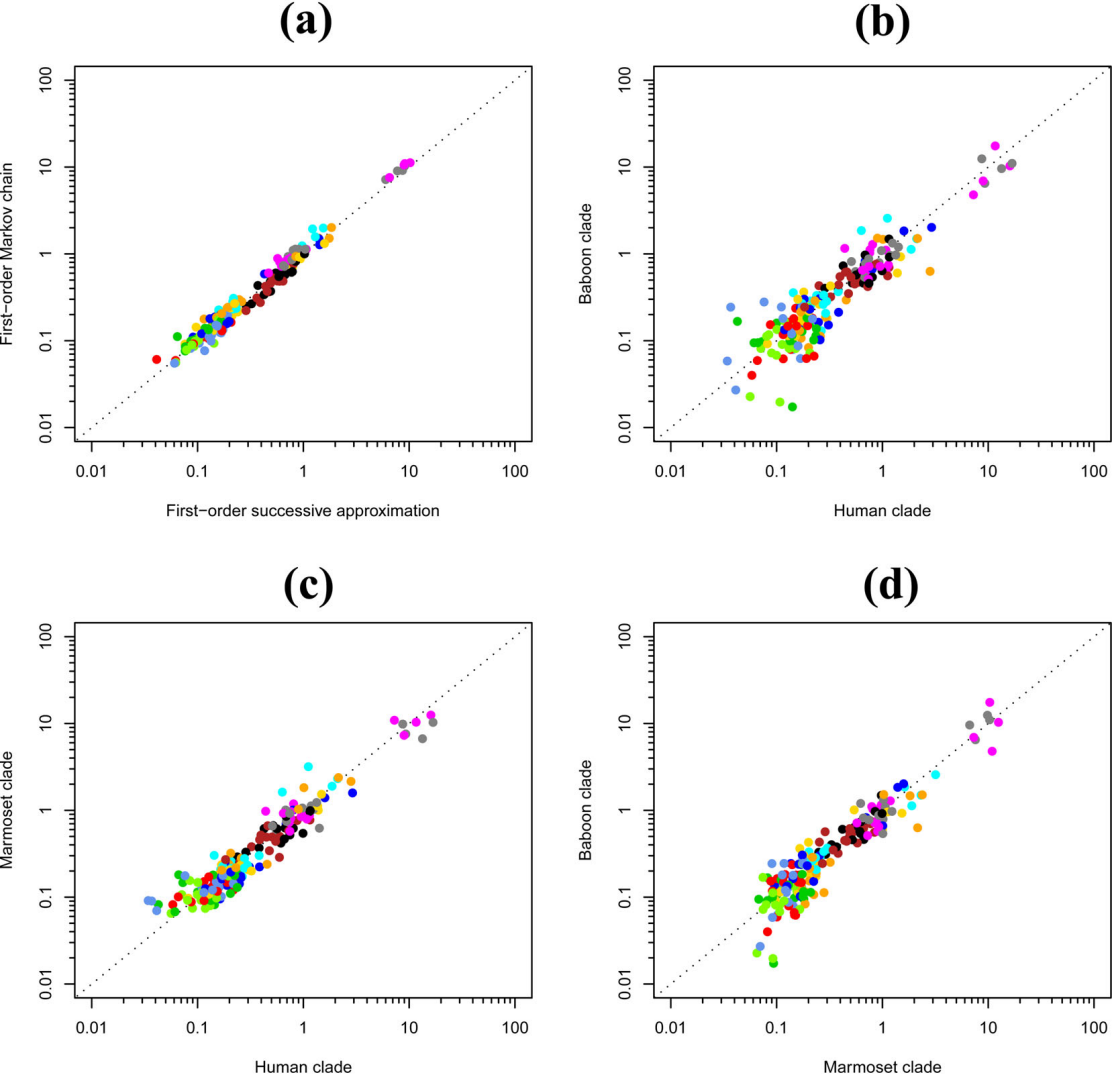
**Ancestral repeats: Comparison of substitution patterns between a coupled and a decoupled root distribution**. (a) Comparison of the substitution patterns of our context-dependent model with context-dependent model frequencies with a decoupled and coupled first-order Markov chain at the ancestral root sequence. (b) Comparison of the substitution patterns between the 'human clade' and 'baboon clade'. (c) Comparison of the substitution patterns between the 'human clade' and 'marmoset clade'. (d) Comparison of the substitution patterns between the 'marmoset clade' and 'baboon clade'. Colour coding is identical to Figures 3, 4 and 5.

Figure 6 (a) shows a comparison of the substitution patterns obtained using our context-dependent model with context-dependent model frequencies, with both a first-order Markov chain and the first-order successive approximation approach to model the ancestral root sequence. There are many differences (but also many similarities) between the two sets of parameter estimates and here we focus on the magnitude of the CpG-effects in both approaches (for a more thorough comparison, we refer to Additional file 1). While Figure 6 (a) shows a clear tendency for the four *r_GA _*and *r_CT _*parameter estimates involved in the CpG-effect to be higher using the (first-order) Markov chain approach, Bayesian p-values (see e.g. [30]) did not show significant differences at the 5% level. Table 4 shows the posterior estimates of the root distribution probabilities using both a first-order Markov chain and a first-order successive approximation approach. Again, Bayesian p-values (see e.g. [30]) indicate which estimates are significantly different at the 5% level.

**Table 4:** Ancestral repeats - Posterior differences in first-order root distribution estimates

	First-order Markov chain			
***X***	***π***_***X|A***_	***π***_***X|C***_	***π***_***X|G***_	***π***_***X|T***_

A	0.3112 [0.3059; 0.3165]	0.1699 [0.1657; 0.1744]	0.2335 [0.2286; 0.2383]	0.2854 [0.2801; 0.2908]
C	0.3583 [0.3515; 0.3653]	0.2464 [0.2403; 0.2527]	0.0345 [0.0308; 0.0382]	0.3607 [0.3539; 0.3678]
G	0.2988 [0.2926; 0.3054]	0.1938 [0.1883; 0.1991]	0.2481 [0.2423; 0.2541]	0.2593 [0.2533; 0.2656]
T	0.2341 [0.2296; 0.2385]	0.1997 [0.1954; 0.2041]	0.2361 [0.2314; 0.2409]	0.3300 [0.3250; 0.3350]

				

	**First-order successive approximation**			

***X***	***π***_***X|A***_	***π***_***X|C***_	***π***_***X|G***_	***π***_***X|T***_

A	0.3176 [0.3117; 0.3237] (11.40%)	0.1689 [0.1641; 0.1738] (76.38%)	0.2241 [0.2186; 0.2295] (1.28%)	0.2894 [0.2835; 0.2953] (32.79%)

C	0.3632 [0.3540; 0.3724] (41.70%)	0.2351 [0.2265; 0.2437] (3.96%)	0.0262 [0.0241; 0.0283] (0.00%)	0.3755 [0.3656; 0.3855] (2.12%)

G	0.3130 [0.3055; 0.3205] (0.62%)	0.1887 [0.1822; 0.1953] (22.97%)	0.2307 [0.2238; 0.2375] (0.07%)	0.2677 [0.2606; 0.2750] (7.94%)

T	0.2399 [0.2343; 0.2455] (11.93%)	0.1901 [0.1846; 0.1957] (0.98%)	0.2333 [0.2276; 0.2390] (44.57%)	0.3367 [0.3302; 0.3433] (11.10%)

Estimates (mean and accompanying 95% credibility interval) for the four sets of base frequencies at the ancestral root, for both first-order Markov chain approach and the successive approximation first-order approach using our context-dependent model with context-dependent model frequencies. Bayesian p-values are reported within brackets with the successive approximation estimates, shedding light on whether the root distribution probabilities are significantly different from one another under both approaches.

The underperformance of the coupled root distribution is quite unexpected as the substitution process along the lineages of the tree should give rise to the root distribution (which is always the case for independent evolutionary models). Lineage-dependent substitution processes, when unaccounted for, can disturb the estimation of the coupled root distribution (but not of the decoupled root distribution). To assess whether the context-dependent substitution process is lineage-dependent, we have divided our observed sequences in three clades: a first clade, called the 'human clade', consists of the sequences for human, chimpanzee, gorilla and orang-utan; a second clade, called the 'baboon clade', consists of the sequences for baboon, macaque and vervet; and a third clade, called the 'marmoset clade', consists of the sequences for marmoset, dusky titi and squirrel monkey. We have performed one MCMC run for each of these three datasets to estimate the context-dependent substitution parameters. Figure 6 (b) to 6 (d) compares these 192 parameters between the three clades using XY plots (if all the parameter means are equal to one another for the two datasets, then the parameter estimates will lie on the diagonal). Large differences can be seen on all three figures, indicating that many context-dependent substitution rates are in fact lineage-dependent, which may explain the underperformance of the coupled root distribution approach.

#### Continuous-time approximation: parameter estimates

**Figure 7:**
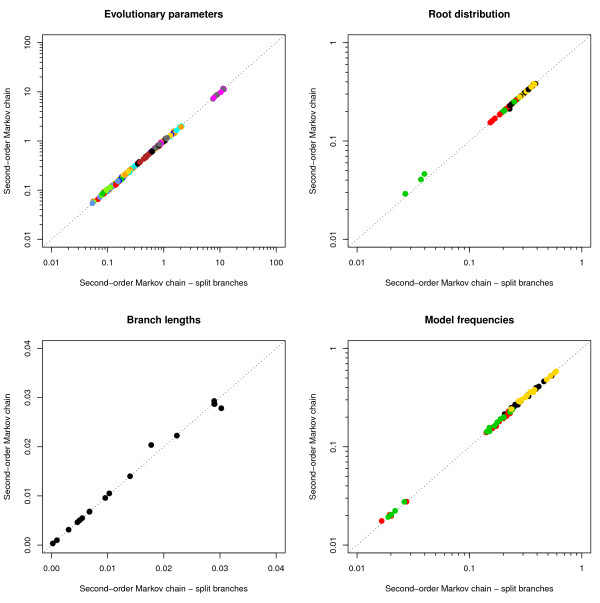
**Ancestral repeats: Comparison of all model parameters with and without splitting branches into multiple parts**. Comparison of evolutionary model estimates, second-order root distribution estimates, branch length estimates and context-dependent model frequencies estimates between an underlying phylogenetic tree with and without branch partitioning. Colour coding for the evolutionary model parameters is identical to Figures 3,4 and 5. The second-order root distribution estimates are coloured as follows: *π_YX|A _*in black, *π_YX|C _*in red, *π_YX|G _*in green and *π_XY|T _*in yellow, with *Y *and *X *the two bases immediately preceding at the 5' side. The model frequencies are coloured as follows: *π_X|A|Y _*in black, *π*_*X|C*|*Y *_in red, *π*_*X|G*|*Y *_in green and *π*_*X|T*|*Y *_in yellow, with *X *the 5' and *Y *the 3' neighbouring base at the ancestral sequence of the branch (i.e. *X*, *Y *∈ {*A*, *C*, *G*, *T*}).

We have performed 100,000 iterations (discarding the first 20,000 iterations as burn-in) to estimate the parameters of our context-dependent evolutionary model with a second-order Markov chain at the root, when the branches are split so that the average substitution rate per time unit is smaller than or equal to 0.005 [3]. We compare all the estimates involved with those of the same model without splitting the branches in Figure 7. The evolutionary parameters show no differences and only minor differences can be seen for the estimates of the model frequencies. No significant differences could be found for the root distribution estimates nor for the branch length estimates. For more details, we refer to Additional file 1.

### Pseudogenes

#### Independent model frequencies: Bayes Factors

**Figure 8:**
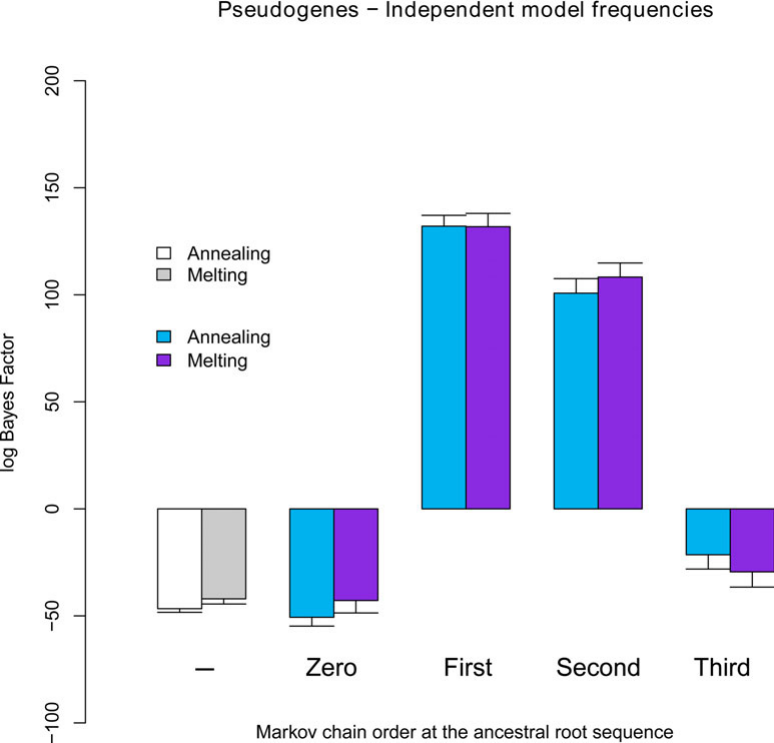
**Pseudogenes: Influence of root sequence distributions on model fit (against independence throughout the entire tree) using independent model frequencies**. Various orders of Markov chains for the ancestral root sequence are tested against the assumption of site-independent evolution throughout the entire tree, revealing that a second-order Markov chain yields the largest increase in model fit vis-à-vis the independent GTR model. The first model comparison (of which the order is indicated by '-') does not assume a separate Markov chain at the ancestral root but uses the independent model frequencies to describe the ancestral root sequence. Both annealing and melting schemes are shown for each model comparison, as well as 95% confidence intervals for both schemes.

We have again compared the performance of the four different Markov chains (zero-, first-, second- and third-order) along the root sequence by calculating the appropriate (log) Bayes Factors. This time, the general time-reversible model (GTR) was used as the reference model. In Figure 8, we calculate the increase in model fit brought about by relaxing the assumption of site-independent evolution at the ancestral root sequence, while assuming a context-dependent model with independent model frequencies (the numerical values are shown in Table S4 in Additional file 1). A first-order Markov chain at the ancestral root sequence yields the largest increase in model fit, outperforming a second-order Markov chain at the ancestral root, which also drastically increases model fit compared to the reference model. All other assumptions in terms of the ancestral root distribution yield negative log Bayes Factors compared to the GTR model and are hence outperformed by a site-independent evolutionary model. The assumption of a third-order Markov chain at the root sequence yields a drastic decrease in model fit compared to the first- and second-order Markov chains, which may be related to the increase in number of parameters.

#### Context-dependent model frequencies: Bayes Factors

**Figure 9:**
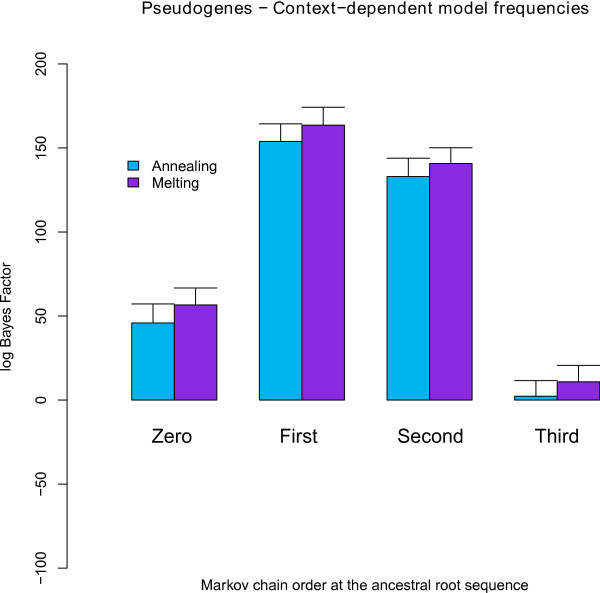
**Pseudogenes: Influence of root sequence distributions on model fit (against independence throughout the entire tree) using context-dependent model frequencies**. Various orders of Markov chains for the ancestral root sequence are tested against the assumption of site-independent evolution throughout the entire tree using the context-dependent model with context-dependent model frequencies, revealing once again that a second-order Markov chain yields the largest increase in model fit vis-à-vis the independent GTR model. Both annealing and melting schemes are shown for each model comparison as well as 95% confidence intervals for both schemes.

Figure 9 shows (log) Bayes Factors for our context-dependent model with context-dependent model frequencies and Markov chains of different orders at the ancestral root sequence (see also Table S5 in Additional file 1). Even when assuming a site-independent distribution at the ancestral root sequence, a drastic increase in model fit is realized through context-dependent model frequencies. As when assuming independent model frequencies, optimal fit is reached when assuming a first-order Markov chain at the ancestral root sequence. Once again, the drastic decrease (both in absolute terms and compared to the first- and second-order Markov chains) of a third-order Markov chain is apparent.

#### Context-dependent model frequencies: root distribution and parameter estimates

As a first-order Markov chain at the ancestral root sequence, combined with context-dependent model frequencies in the evolutionary model, yields the largest increase in model fit, we here report the root distribution and parameter estimates for this model. The first-order ancestral root distribution estimates can be seen in Table 5, from which the decreased probability of observing a G at a given site when its preceding site is a G is immediately apparent due to the 5-methylcytosine deamination process (i.e., the CpG effect). This is in accordance with the results for the ancestral repeats dataset, although in that dataset a second-order Markov chain was used. These differences once again demonstrate the importance of relaxing the assumption of independent model frequencies.

**Table 5:** Pseudogenes - First-order root distribution estimates

	Root			
***X***	***π***_***X|A***_	***π***_***X|C***_	***π***_***X|G***_	***π***_***X|T***_

A	0.3231 [0.3025; 0.3444]	0.1659 [0.1492; 0.1833]	0.2315 [0.2124; 0.2508]	0.2795 [0.2589; 0.3005]
C	0.3440 [0.3160; 0.3727]	0.2243 [0.2003; 0.2497]	0.0361 [0.0236; 0.0509]	0.3956 [0.3678; 0.4243]
G	0.3321 [0.3060; 0.3591]	0.1730 [0.1526; 0.1949]	0.2273 [0.2041; 0.2511]	0.2676 [0.2430; 0.2925]
T	0.2486 [0.2290; 0.2686]	0.1886 [0.1713; 0.2068]	0.2547 [0.2350; 0.2748]	0.3081 [0.2869; 0.3291]

Estimates (mean and accompanying 95% credibility interval) for the sixteen sets of base frequencies at the ancestral root sequence under the context-dependent model with context-dependent model frequencies. The probabilities are grouped by the identity of the immediately preceding site.

**Figure 10:**
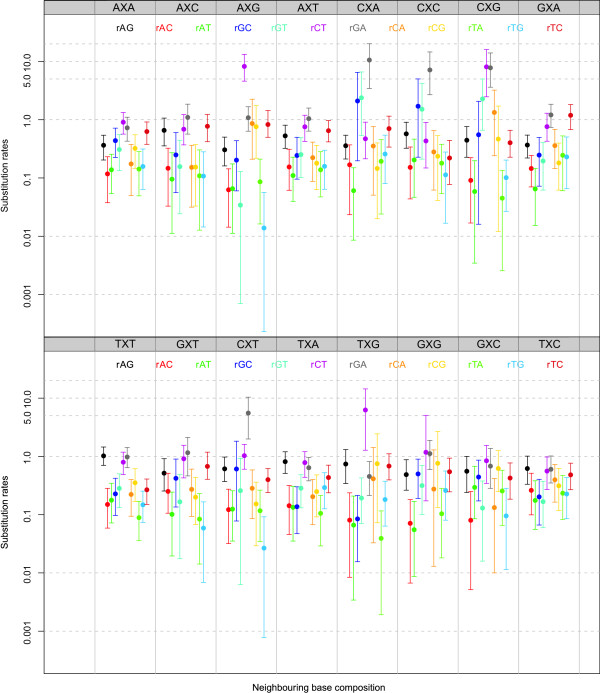
**Pseudogenes: Substitution parameters for context-dependent model frequencies and a first-order Markov chain at the root**. Substitution parameter estimates (mean and 95% credibility intervals) for each of the twelve entries of the general time-reversible model in each of the sixteen neighbouring base combination using context-dependent model frequencies and a first-order Markov chain to specify the ancestral root distribution. Note that the log-scale is used for the y-axis.

Figure 10 shows all the substitution rates of our context-dependent model using context-dependent model frequencies, assuming a first-order Markov chain to describe the sequence composition of the ancestral root. As for the ancestral repeats dataset, a large variation in substitution rates across different neighbouring base combinations can be observed. Given the presence of the CpG-methylation-deamination process in mammals, we once again focus on the substitution rates from C to T (*r_CT_*) in the AXG, CXG, GXG and TXG contexts as well as their compensating substitution rates from G to A (*r_GA_*) in the respective CXT, CXG, CXC and CXA contexts. The mean substitution rates *r_CT _*are 8.22, 8.07, 1.18 and 6.25 for the AXG, CXG, GXG and TXG contexts, respectively, and 5.54, 7.77, 7.14 and 10.56 for the compensating substitution rates *r_GA _*in the CXT, CXG, CXC and CXA contexts, respectively. Note that the context-dependent model does not enforce compensatory mutations to be equal to one another. Immediately apparent is the decreased *r_CT _*substitution rate in the GXG context, which we can only attribute to a lack of sites in this context, and the increased *r_GA _*substitution rate in the CXA context. All context-dependent substitution rates involved in the CpG-methylation-deamination process (except for the *r_GA _*estimate in the CXA context) are lower than those estimated for the optimal model for the ancestral repeats dataset.

In Table S6 in Additional file 1, we report the estimates for the context-dependent model frequencies for our optimal model for the pseudogenes dataset. The decreased *r_CT _*substitution rate in the GXG context mentioned above is reflected in the first set of frequency estimates related to the CpG-methylation-deamination process: *π_A|C|G_*, *π_C|C|G_*, *π_G|C|G_*, *π_T|C|G _*, where it can be seen that the *π_G|C|G _*estimate is much higher than the estimates for *π_A|C|G_*, *π_C|C|G _*and *π_T|C|G_*. The second set of CpG-related frequency estimates however shows fairly equal estimates for *π_C|G|A_*, *π_C|G|C_*, *π_C|G|G_*, *π_C|G|T_*.

## Discussion

In this paper we have demonstrated the importance of modelling non-independent root distributions at the ancestral root sequence in a phylogenetic tree. This completes the dependency scheme across the entire tree so that the evolution at every nucleotide, ancestral or observed, is allowed to depend on other nucleotides. We have found that the use of a second-order Markov chain at the ancestral root sequence results in the largest increase in terms of model fit for a large dataset of primate ancestral repeat sequences. For a smaller dataset of primate pseudogenes, we have found that a first-order Markov chain at the ancestral root sequence yields the largest increase in model fit. We have calculated the differences in model fit using computationally demanding (see Additional file 1 for computation time requirements), but accurate Bayes Factor calculations and report drastic increases in model fit by modelling context-dependence, especially compared to other evolutionary assumptions such as among-site rate variation. Indeed, as shown in previous work [18], the increase in model fit brought about by assuming among-site rate variation equals about 355 log units, which is drastically lower than the more than 7.000 log units increase reported here. Further, for the pseudogenes dataset, the increase in model fit brought about by the optimal context-dependent model and root distribution equals about 150 log units, compared to an increase in model fit for among-site rate variation with a bidirectional mean log Bayes Factor of 5.4 units (annealing: [5.5; 5.7]; melting: [5.0; 5.2]), corresponding to the findings of Yang [14]. As far as we know and given the known presence of CpG-effects in our dataset, context-dependent models seem to be the best choice for increasing the evolutionary model's realism.

Our approach is probably best compared to that of Hwang and Green [3], although we have not employed branch-specific context-dependent models due to the fact that our dataset consisted only of primate sequences. We have shown, using careful and computationally intensive model selection, that the choice of the ancestral root distribution heavily influences the substitution rates for the CpG-methylation-deamination process and that first- and second-order Markov chains at the root sequence, independent of the context-dependent evolutionary model, are better fit to describe the data than a first-order approximation coupled to the context-dependent evolutionary model. Hwang and Green [3] do not employ a model selection approach in their study on a branch-specific context-dependent model.

Even though Hwang and Green [3] use more data (longer sequences and more species), statistical support for their modelling choices in terms of observed increase in model fit, which we have shown here to be a crucial aspect of examining complex evolutionary models, should be used to further strengthen their findings [23]. Given the complexity of branch-specific context-dependent models, model comparison is however seriously hampered by the immense computational demands. Our estimates of the substitution rates for the CpG-effect seem to be lower than those reported by Hwang and Green [3], although the authors do not report the mean estimates so only a visual comparison is possible. The *r_CT _*and *r_TC _*substitution rates are generally more elevated in the work of Hwang and Green [3], but this could be specific to the untranscribed regions the authors have investigated.

We have also shown that the discrete approximation to the continuous-time Markov substitution process, as used by Hwang and Green [3], yields a significant increase in model fit in terms of the log Bayes Factors calculated. As the evolutionary model parameters were unaffected by partitioning the branches where appropriate, the branch partitioning approach itself is responsible for more accurately modelling the ancestral states throughout the underlying phylogenetic tree. As mentioned in the Methods section, we have split each branch into two or more parts of equal length such that the average substitution rate per time unit is smaller than or equal to 0.005 [3]. However, by no means does this approach guarantee that the optimal number of branch partitions are used and that the corresponding increase in model fit over an independent evolutionary model is maximized. Further research on such discrete approximations is reported in the work of de Koning et al. [29].

The design and study of context-dependent evolutionary models is also of interest when studying coding sequence evolution, as shown by recent publications on mutation-selection models (see e.g. [33-35]). Indeed, while current mutation-selection models use the general-time reversible model (GTR) for modelling mutation bias (see e.g. [34,35]), the context-dependent model presented in this manuscript may be better suited to model mutation bias at either one of the three codon positions.

## Conclusions

Designing accurate context-dependent models is a complex process, with many different assumptions that require testing using an accurate procedure for model testing, which are computationally very demanding. Not only the context-dependent evolutionary model itself requires estimation and evaluation, we have also shown that the choice of an adequate ancestral root distribution is essential to accurately estimate the model's parameters. Moreover, we have also shown that the choice of an ancestral root distribution, changes across different datasets. In other words, there is no single context-dependent model or ancestral root distribution that can be argued to work well for a given dataset, without having to actually perform a series of model comparisons.

We have shown in this paper that context-dependent models are very useful when analyzing primate sequences, due to the presence of the CpG-methylation-deamination process. Since this is a process known to occur in mammalian evolution, context-dependent evolutionary models should also prove useful in the analysis of non-primate mammalian datasets. Even though the context-dependent models presented in this paper greatly increase model fit over independent models in the two datasets studied, additional work on context-dependent evolutionary models is required. For example, we have shown that an ancestral root distribution coupled to the context-dependent model does not improve model fit as much as a decoupled ancestral root distribution does. While we have provided a possible explanation for this result, additional work is required to clarify this issue.

## Software availability

Additional file 5 contains the necessary software routines, evolutionary models, ancestral root distributions and several example input files for the software to reproduce the various results presented in this paper. More information on how to use the software can be found in Additional file 1.

## Authors' contributions

GB initiated the study, designed the context-dependent evolutionary model, implemented the Markov chain root distribution approach, co-designed the coupled root distribution approach, performed all the analyses, programmed the software routines and wrote a first complete version of the manuscript. YVdP contributed biological expertise to the analyses and edited the manuscript. SV contributed statistical expertise to the analyses, co-designed the coupled root distribution approach and edited the manuscript. All authors read and approved the final manuscript.

## Supplementary Material

Additional file 1 **File containing supplementary material and information that was not included in the main document**. Click here for file

Additional file 2 **File containing supplementary figure S1**. Encapsulated PostScript figure. Click here for file

Additional file 3 **File containing supplementary figure S2**. Encapsulated PostScript figure. Click here for file

Additional file 4 **File containing supplementary figure S3**. Encapsulated PostScript figure. Click here for file

Additional file 5 **File containing software classes, alignments used and example input files**. Click here for file
