# Supplementary material for: Modelling the ancestral sequence distribution and model frequencies in context-dependent models for primate non-coding sequences
Source: BMC Evol Biol. 2010 Aug 10;10:244. doi: 10.1186/1471-2148-10-244 (PMC2928787; doi:10.1186/1471-2148-10-244)
Supplement: Additional file 1 — File containing supplementary material and information that was not included in the main document. [file 1471-2148-10-244-S1.DOC]

**Ancestral repeats - Simulation studies**

We have first simulated the ancestral root sequence using the posterior means reported for the second-order Markov chain. The sequence length was set equal to that of our dataset, i.e. 114,726 sites for each sequence and the underlying phylogenetic tree was identical to the one used in the data analysis. The branch lengths were set to equal the mean branch lengths inferred under the optimal model reported in this paper. We then simulated the 10 observed data sequences using the posterior parameter means of our context-dependent evolutionary model and repeated this process 20 times, resulting in 20 different simulated datasets.

The second series of simulations focuses on an ancestral root distribution coupled to the context-dependent evolutionary model. Given that the context-dependent model assumes that the evolution of a given site depends on its two immediate flanking sites, only a first-order dependency pattern at the ancestral root can reliably be calculated from the model using a two-cluster approximation (as explained above). The ancestral root sequence was first simulated using the posterior means of the root distribution estimates, after which the 10 observed data sequences were evolved from this root distribution using the posterior parameter means of our context-dependent evolutionary model. Again, we repeated this process 20 times, resulting in 20 different simulated datasets. The same settings for sequence length, tree topology and branch lengths as in the first series were used.

**Ancestral Repeats - Simulation study: decoupled second-order Markov chain at the root**

As a second-order Markov chain at the ancestral root sequence along with context-dependent model frequencies has been assessed as the optimal model (see Figure 2), we have performed a simulation experiment to verify the accuracy of the reported results. 20 simulated datasets were created (see the Methods section) and the aforementioned Bayesian MCMC approach was then used to re-estimate the parameters from all 20 datasets (calculating 100,000 iterations and discarding the first 20,000 as the burn-in sequence). Figure S1 (see Additional file 2) shows the error distributions for the 96 evolutionary parameters, the 64 root distribution probabilities, the 18 branch lengths and the 64 model frequencies which stem from the evolutionary model. The normalized errors were computed by dividing the difference between the true parameter value and the average of the posterior means by the standard deviation of the posterior means (similar to Hwang and Green, 2004; but using 20 simulations instead of a single run). For each of the four parameter types, the error distribution is approximately normal around zero, indicating that our MCMC approach (with the given prior settings) is able to reliably estimate parameter values from such a large dataset.

**Ancestral repeats - Simulation study: coupled first-order Markov chain at the root**

To test whether our two-cluster approximation has been performed correctly, we have performed a simulation experiment for this approach as well. Again, 20 simulated datasets were created and our Bayesian MCMC approach was used to re-estimate the parameters from all 20 datasets (calculating 100,000 iterations and discarding the first 20,000 as the burn-in sequence). Figure S2 (see Additional file 3) shows the error distributions for the 96 evolutionary parameters, the 16 root distribution probabilities, the 18 branch lengths and the 64 model frequencies which stem from the evolutionary model. The normalized errors were computed by dividing the difference between the true parameter value and the average of all means by the standard deviation of the means (similar to Hwang and Green [3]; but using 20 simulations instead of a single run). For each of the four parameter types, the error distribution is approximately normal around zero, indicating that our results concerning the coupled root distribution are indeed reliable.

**Dirichlet prior distribution**

The mean of the prior distribution for each rate of the GTR model family is simply the value of the corresponding Dirichlet parameter divided by the sum of all six Dirichlet parameters. The variance of each GTR rate around this mean is inversely related to the sum of the Dirichlet parameters. Thus, a GTR model with a Dirichlet(1, 1, 1, 1, 1, 1) prior and a model with a Dirichlet(1000,1000, 1000, 1000, 1000, 1000) prior both have an expectation of equal rates for all substitution types, but the latter prior heavily penalizes models of evolution in which the rates are not nearly equal. An extensive discussion on using a Dirichlet prior distribution for the GTR model can be seen in the work of Zwickl and Holder [22].

**Context-dependent model frequencies: Bayes Factors**

Note that both annealing and melting Bayes Factor calculations in Supplementary Table S2 show a large overlap for the zero- and first-order root distributions, but not for the second-order and third-order root distributions. Even though the width of the confidence intervals is less than 1% of the mean log Bayes Factor estimate, a split-calculation approach [18] was adopted to further improve accuracy, allowing for 3.6 times the number of iterations used for the results in Figure 2. This results in a log Bayes Factor of 7218.01 ([7200.34 ; 7235.68]) for the annealing scheme and a log Bayes Factor of 7224.94 ([7207.16 ; 7242.71]) for the melting scheme, yielding a bidirectional mean log Bayes Factor of 7221.47 log units, compared to the independent GTR model. Given the drastic differences in model fit between the different models, this does not alter our previous conclusions.

**Third-order Markov chains**

One way to reduce the penalty for the increase in parameters is to cluster similar sets of model frequencies together, thereby reducing the number of parameters as well as the amount of noise. Such an approach would be similar to the clustering of neighbouring base compositions in Baele et al. [18] (see Figure S3 in Additional file 4). The top four rows show sixteen different cluster plots for the context-dependent model with context-dependent model frequencies, while the bottom two rows show the five cluster plots involved in the CpG-methylation-deamination process for the context-dependent model assuming independent model frequencies (the other 11 cluster plots were very similar for both context-dependent models; data not shown).

We have tried to represent the different cluster plots on the same scale for both x-axis (First PC; representing the first principal component) and y-axis (Second PC; representing the second principal component) but the five cluster plots containing neighbouring base combinations that are involved in the CpG-methylation-deamination process, needed to be drawn to a different scale. The identities of the preceding sites which are involved in CpG-effects are denoted X-C-G, with X the left-most preceding site on which a given site depends, X-G-A, X-G-C, X-G-G and X-G-T. The X-C-G combination, with X one out of A, C, G or T, is bound to be underrepresented due to the CpG-effect as most CpG dinucleotides will have mutated to either TpG or CpA dinucleotides. As there is only few data available to estimate the corresponding base frequencies in the ancestral root sequence, there will be much variation in the inferred parameters. The same goes for the other four mentioned combinations (X-G-A, X-G-C, X-G-G and X-G-T), where an increase in variability is clearly noticeable when the left-most nucleotide is a C (plotted in red in Figure S3). However, this increase in variability heavily depends on the assumption for the model frequencies. Indeed, as can be seen from the bottom two rows in Figure S3, when assuming a single set of independent model frequencies in our context-dependent model, the variability of the root distribution probabilities increases drastically in the five cluster plots involved in the CpG-methylation-deamination process. In other words, when allowing for context-dependent model frequencies, the root distribution probabilities can be estimated more accurately than when assuming a set of independent model frequencies. This again shows that the assumption of modelling a Markov chain to describe the root distribution is not independent from the evolutionary model used.

**Coupled root distribution and context-dependent evolutionary model: parameter estimates**

There are many differences (but also many similarities) between the two sets of parameter estimates. Of the 192 context-dependent evolutionary parameters, 32 (or 16.7%) were significantly different at the 5% level between both approaches. No significant differences were found for the $r_{AC}$, $r_{GC}$ and $r_{TA}$ parameters, while the $r_{AT}$ parameter was found to differ significantly in the CXG context and the $r_{CG}$ parameter in the CXT context. Further, the $r_{GT}$ and $r_{TG}$ parameters differed significantly in the CXG and GXT contexts and the $r_{CA}$ parameter in the CXT and TXT contexts. The remaining parameters differed significantly in five or more contexts: the $r_{AG}$ parameter in the AXG, GXA, GXC, GXG and GXT contexts; the $r_{GA}$ parameter in the AXA, AXG, AXT, GXA, GXC, GXG and GXT contexts; the $r_{CT}$ parameter in the CXA, CXC, TXC, CXT, GXT and TXT contexts; and the $r_{TC}$ parameter in the CXA, CXG, CXT, TXA, TXC and TXT contexts.

In Table 4, the posterior estimates of the root distribution probabilities using both a first-order Markov chain and a first-order successive approximation approach are compared using Bayesian p-values. When A or T is the preceding base, only one estimate was found to differ significantly, while two significant differences were found with G as the preceding base and three with C as the preceding base. The most drastic difference was found in the estimate of $\pi_{C\left|G\right.}$, which is directly linked to the presence of CpG-effects. Note that the 95% credibility interval is about twice as wide for the $\pi_{C\left|G\right.}$ estimate when estimated using a first-order Markov chain, compared to the successive approximation method.

We would also like to note that the context-dependent substitution patterns are highly similar when using either a first-order or a second-order Markov chain at the ancestral root. From two independent MCMC runs of 100,000 iterations (one for the first-order and one for the second-order Markov chain, with the same underlying model), we have calculated Bayesian p-values (see e.g. [30]) for each of the 192 parameter estimates. None of these estimates could be shown to be significantly different from one another at the 5% significance level. Independence at the ancestral root sequence thus prohibits accurate estimation of the CpG-effect(s) while modelling first-order or second-order dependencies both yield highly similar results.

**Continuous-time approximation: parameter estimates**

The root distribution estimates show small differences in five parameters, i.e. the estimates of $\pi_{G}$ when the two preceding bases are AC (0.040 vs. 0.046; Bayesian p-value: 0.27), CC (0.037 vs. 0.041; Bayesian p-value: 0.49), GC (0.027 vs. 0.029; Bayesian p-value: 0.68) and TC (0.027 vs. 0.029; Bayesian p-value: 0.57) and the estimate of $\pi_{A}$ when the two preceding bases are CG (0.228 vs. 0.212; Bayesian p-value: 0.58). In other words, none of the observed differences in the root distribution are significant at the 5% significance level. The branch length estimates show two minor differences, i.e. for the two branches connected to the ancestral root nodes. Neither the first root branch (connecting the root to the ‘human and baboon clades’) estimates (0.018 vs. 0.020; Bayesian p-value: 0.14) nor the second root branch (connecting the root to the ‘marmoset clade’) estimates (0.030 vs. 0.028; Bayesian p-value: 0.19) show significant differences at the 5% significance level.

**Calculations: computation time requirements**

Each model comparison (i.e. each Bayes Factor calculation) for the ancestral repeats dataset, that did not require split branches, took about 7 days on 40 processors, except for the more demanding recalculation of the optimal model, which took about 7 days on 144 processors. The model comparison with split branches, resulting in 52 branch parts instead of 18 when the branches are not split, took about 3 times as long. Further, each MCMC run to acquire parameter estimates took about 7 days on a single processor/core, both on the real and the simulated datasets (except for the MCMC run with split branches which took about three times as long). Each model comparison for the pseudogenes dataset took about 1 day on 10 processors, with a single MCMC run to acquire parameter estimates taking only 4 hours. This leads to a total of over 24 years of computation time required to perform the necessary parameter estimations, simulations and model selection calculations reported in this paper (if calculations were to run on a single processor/core).

**Figure S1 (FigureS1.eps) – Error distribution for the simulation study with a decoupled root distribution.**Error distribution for 96 evolutionary parameters, 64 decoupled second-order root distribution frequencies, 18 branch lengths and 64 model frequencies, obtained from 20 (different) simulated datasets. Our MCMC approach was used to estimate the parameter estimates from each of these simulated datasets and normalized error estimates were obtained by subtracting the true parameter value from the mean of the posterior means of the parameters and standardizing afterwards. The error distribution for each of the 4 parameter types is approximately normal with mean zero, which indicates that our MCMC approach is able to reliably estimate parameter values and is also able to overthrow (already weak) prior assumptions.

**Figure S2 (FigureS2.eps) - Error distribution for the simulation study with a coupled root distribution.**
Error distribution for 96 evolutionary parameters, 16 coupled root distribution frequencies, 18 branch lengths and 64 model frequencies, obtained from 20 (different) simulated datasets. Our MCMC approach was used to estimate the parameter estimates from each of these simulated datasets and normalized error estimates were obtained by subtracting the true parameter value from the mean of the posterior means of the parameters and standardizing afterwards. The error distribution for each of the 4 parameter types is approximately normal with mean zero, which indicates that our MCMC approach is able to reliably estimate parameter values and is also able to overthrow (already weak) prior assumptions.

**Figure S3 (FigureS3.eps) – Ancestral repeats: Four-dimensional clustering of third-order root frequencies.**
A four-dimensional principal component clustering of third-order root frequencies for the context-dependent model with independent model frequencies (top four rows) and with context-dependent model frequencies (bottom two rows; only relevant differences with independent model frequencies shown). X is the left-most nucleotide on which a given nucleotide depends and for which the following colour scheme is applied: A: blue; C: red; G: green; T: grey. This clustering comparison reveals a drastic decrease in variability for those neighbouring base combinations involved in the CpG-methylation-deamination process (i.e. X-C-G, X-G-A, X-G-C, X-G-G and X-G-T) and hence illustrates the importance of assuming context-dependent model frequencies.

**Table S1: Ancestral repeats - Influence of various root sequence distributions on model fit (against independence throughout the entire tree) using independent model frequencies**

| Root | Parameters | Annealing | Melting | Log BF |
| --- | --- | --- | --- | --- |
| - | 90 | [623.20 ; 638.25] | [645.53 ; 661.86] | 642.15 |
| 0 | 94 | [737.26 ; 767.61] | [758.74 ; 785.10] | 762.18 |
| 1 | 106 | [5286.53 ; 5317.74] | [5316.63 ; 5350.89] | 5317.95 |
| 2 | 154 | [5573.44 ; 5608.37] | [5593.45 ; 5630.29] | 5601.39 |
| 3 | 346 | [5440.41 ; 5479.08] | [5470.62 ; 5510.13] | 5475.06 |

Various orders of Markov chains for the ancestral root sequence are tested against the assumption of site-independent evolution throughout the entire tree. The first column shows the order of the Markov chain at the ancestral root sequence (the first comparison assumes that the base frequencies are stationary); the second column shows the number of additional parameters required vis-à-vis the independent GTR model; the third and fourth column show the 95% confidence interval for the log Bayes Factor in both annealing and melting schemes of thermodynamic integration; the fifth column shows the mean log Bayes Factor calculated from the third and fourth column vis-à-vis the independent GTR model.

**Table S2: Ancestral repeats - Influence of various root sequence distributions on model fit (against independence throughout the entire tree) using context-dependent model frequencies**

| Root | Parameters | Annealing | Melting | Log BF |
| --- | --- | --- | --- | --- |
| 0 | 154 | [4752.71 ; 4820.51] | [4748.88 ; 4812.20] | 4783.57 |
| 1 | 166 | [6903.37 ; 6947.97] | [6906.96 ; 6954.79] | 6928.27 |
| 2 | 214 | [7181.42 ; 7228.09] | [7225.90 ; 7277.09] | 7228.12 |
| 3 | 406 | [7065.51 ; 7114.34] | [7107.26 ; 7160.81] | 7111.98 |

Various orders of Markov chains for the ancestral root sequence are tested against the assumption of site-independent evolution throughout the entire tree using the context-dependent model with context-dependent model frequencies. The first column shows the order of the Markov chain at the ancestral root sequence; the second column shows the number of additional parameters required vis-à-vis the independent GTR model; the third and fourth column show the confidence interval for the log Bayes Factor in both annealing and melting schemes of thermodynamic integration; the fifth column shows the mean log Bayes Factor calculated from the third and fourth column vis-à-vis the independent GTR model.

**Table S3: Ancestral repeats - Context-dependent model frequencies estimates**

| $YX$ | $\pi_{Y\left\vert A\left\vert X\right.\right.}$ | $\pi_{Y\left\vert C\right.\left\vert X\right.}$ | $\pi_{Y\left\vert G\right.\left\vert X\right.}$ | $\pi_{Y\left\vert T\right.\left\vert X\right.}$ |
| --- | --- | --- | --- | --- |
| AA | 0.3269  [0.2944 ; 0.3606] | 0.2290  [0.2074 ; 0.2510] | 0.1433  [0.1253 ; 0.1633] | 0.3008  [0.2713 ; 0.3326] |
| CA | 0.4100  [0.3495 ; 0.4740] | 0.2047  [0.1729 ; 0.2379] | 0.0275  [0.0227 ; 0.0332] | 0.3578  [0.3046 ; 0.4097] |
| GA | 0.3844  [0.3366 ; 0.4308] | 0.2178  [0.1837 ; 0.2543] | 0.1561  [0.1286 ; 0.1866] | 0.2417  [0.2033 ; 0.2861] |
| TA | 0.2799  [0.2507 ; 0.3094] | 0.1944  [0.1713 ; 0.2198] | 0.2398  [0.2130 ; 0.2667] | 0.2859  [0.2563 ; 0.3169] |
| AC | 0.3341  [0.2830 ; 0.3855] | 0.1490  [0.1252 ; 0.1761] | 0.1986  [0.1680 ; 0.2314] | 0.3184  [0.2676 ; 0.3743] |
| CC | 0.2952  [0.2366 ; 0.3638] | 0.1575  [0.1277 ; 0.1909] | 0.0223  [0.0181 ; 0.0275] | 0.5251  [0.4638 ; 0.5784] |
| GC | 0.3345  [0.2805 ; 0.3925] | 0.1619  [0.1319 ; 0.1947] | 0.1773  [0.1498 ; 0.2080] | 0.3263  [0.2715 ; 0.3800] |
| TC | 0.2495  [0.2144 ; 0.2860] | 0.1541  [0.1298 ; 0.1803] | 0.2384  [0.2073 ; 0.2723] | 0.3580  [0.3182 ; 0.3996] |
| AG | 0.5260  [0.4809 ; 0.5647] | 0.0198  [0.0171 ; 0.0228] | 0.1630  [0.1380 ; 0.1912] | 0.2911  [0.2509 ; 0.3403] |
| CG | 0.3968  [0.2878 ; 0.5493] | 0.0176  [0.0140 ; 0.0217] | 0.0204  [0.0162 ; 0.0253] | 0.5652  [0.4121 ; 0.6742] |
| GG | 0.4622  [0.4055 ; 0.5142] | 0.0203  [0.0169 ; 0.0244] | 0.1421  [0.1198 ; 0.1673] | 0.3753  [0.3180 ; 0.4382] |
| TG | 0.2887  [0.2410 ; 0.3391] | 0.0276  [0.0231 ; 0.0328] | 0.2008  [0.1710 ; 0.2320] | 0.4829  [0.4210 ; 0.5434] |
| AT | 0.2672  [0.2212 ; 0.3165] | 0.2105  [0.1771 ; 0.2479] | 0.1911  [0.1597 ; 0.2257] | 0.3313  [0.2809 ; 0.3829] |
| CT | 0.2147  [0.1792 ; 0.2563] | 0.1805  [0.1456 ; 0.2193] | 0.0193  [0.0163 ; 0.0229] | 0.5855  [0.5381 ; 0.6271] |
| GT | 0.2686  [0.2234 ; 0.3186] | 0.2186  [0.1820 ; 0.2584] | 0.1454  [0.1206 ; 0.1741] | 0.3673  [0.3182 ; 0.4162] |
| TT | 0.2554  [0.2270 ; 0.2875] | 0.1397  [0.1198 ; 0.1612] | 0.2255  [0.2018 ; 0.2507] | 0.3793  [0.3433 ; 0.4156] |

Estimates (mean and accompanying 95% credibility interval) for the sixteen sets of base frequencies throughout the tree under the context-dependent model with context-dependent model frequencies. The probabilities are grouped by the identity of the immediate preceding site. Note that the model frequencies used in the remainder of the tree are dependent upon the base’s two immediate flanking bases.

**Table S4: Pseudogenes - Influence of various root sequence distributions on model fit (against independence throughout the entire tree) using independent model frequencies**

| Root | Parameters | Annealing | Melting | Log BF |
| --- | --- | --- | --- | --- |
| - | 90 | [-48.33 ; -44.91] | [-44.45 ; -39.60] | -44.32 |
| 0 | 94 | [-54.77 ; -46.53] | [-48.62 ; -37.01] | -46.73 |
| 1 | 106 | [127.00 ; 137.12] | [125.60 ; 138.02] | 131.94 |
| 2 | 154 | [93.98 ; 107.55] | [101.68 ; 114.86] | 104.52 |
| 3 | 346 | [-28.11 ; -14.78] | [-36.57 ; -22.29] | -25.44 |

Various orders of Markov chains for the ancestral root sequence are tested against the assumption of site-independent evolution throughout the entire tree. The first column shows the order of the Markov chain at the ancestral root sequence (the first comparison assumes that the base frequencies are stationary); the second column shows the number of additional parameters required vis-à-vis the independent GTR model; the third and fourth column show the 95% confidence interval for the log Bayes Factor in both annealing and melting schemes of thermodynamic integration; the fifth column shows the mean log Bayes Factor calculated from the third and fourth column vis-à-vis the independent GTR model.

**Table S5: Pseudogenes - Influence of various root sequence distributions on model fit (against independence throughout the entire tree) using context-dependent model frequencies**

| Root | Parameters | Annealing | Melting | Log BF |
| --- | --- | --- | --- | --- |
| 0 | 154 | [34.50 ; 57.21] | [46.51 ; 66.70] | 51.23 |
| 1 | 166 | [143.46 ; 164.35] | [152.88 ; 174.25] | 158.73 |
| 2 | 214 | [122.05 ; 143.92] | [131.33 ; 150.13] | 136.86 |
| 3 | 406 | [-7.04 ; 11.61] | [1.07 ; 20.60] | 6.56 |

Various orders of Markov chains for the ancestral root sequence are tested against the assumption of site-independent evolution throughout the entire tree using the context-dependent model with context-dependent model frequencies. The first column shows the order of the Markov chain at the ancestral root sequence; the second column shows the number of additional parameters required vis-à-vis the independent GTR model; the third and fourth column show the confidence interval for the log Bayes Factor in both annealing and melting schemes of thermodynamic integration; the fifth column shows the mean log Bayes Factor calculated from the third and fourth column vis-à-vis the independent GTR model.

**Table S6: Pseudogenes - Context-dependent model frequencies estimates**

| $YX$ | $\pi_{Y\left\vert A\left\vert X\right.\right.}$ | $\pi_{Y\left\vert C\right.\left\vert X\right.}$ | $\pi_{Y\left\vert G\right.\left\vert X\right.}$ | $\pi_{Y\left\vert T\right.\left\vert X\right.}$ |
| --- | --- | --- | --- | --- |
| AA | 0.3131  [0.2035 ; 0.4211] | 0.2164  [0.1420 ; 0.3034] | 0.1584  [0.0930 ; 0.2300] | 0.3121  [0.2089 ; 0.4243] |
| CA | 0.5403  [0.2963 ; 0.7128] | 0.2622  [0.1339 ; 0.4493] | 0.0212  [0.0093 ; 0.0516] | 0.1763  [0.0833 ; 0.3677] |
| GA | 0.4965  [0.3428 ; 0.6314] | 0.2131  [0.1086 ; 0.3375] | 0.1553  [0.0820 ; 0.2603] | 0.1351  [0.0681 ; 0.2415] |
| TA | 0.2380  [0.1475 ; 0.3480] | 0.1652  [0.0914 ; 0.2567] | 0.3014  [0.1973 ; 0.4224] | 0.2954  [0.1863 ; 0.4148] |
| AC | 0.2858  [0.1585 ; 0.4422] | 0.2850  [0.1367 ; 0.4495] | 0.1786  [0.0665 ; 0.3270] | 0.2507  [0.1198 ; 0.3947] |
| CC | 0.3610  [0.2358 ; 0.5046] | 0.2095  [0.0897 ; 0.3760] | 0.0326  [0.0140 ; 0.0676] | 0.3970  [0.2248 ; 0.5597] |
| GC | 0.2798  [0.1322 ; 0.4797] | 0.1660  [0.0781 ; 0.2813] | 0.2302  [0.1210 ; 0.3492] | 0.3240  [0.1649 ; 0.5059] |
| TC | 0.2916  [0.1761 ; 0.4503] | 0.1914  [0.1030 ; 0.3060] | 0.2993  [0.1773 ; 0.4369] | 0.2176  [0.1250 ; 0.3512] |
| AG | 0.4705  [0.2523 ; 0.6517] | 0.0355  [0.0195 ; 0.0585] | 0.1347  [0.0574 ; 0.2345] | 0.3593  [0.1797 ; 0.6102] |
| CG | 0.4165  [0.1857 ; 0.6431] | 0.0301  [0.0115 ; 0.0666] | 0.0244  [0.0100 ; 0.0472] | 0.5290  [0.2911 ; 0.7663] |
| GG | 0.4157  [0.2339 ; 0.6150] | 0.1758  [0.0268 ; 0.3898] | 0.1815  [0.0974 ; 0.2961] | 0.2271  [0.1071 ; 0.4464] |
| TG | 0.2248  [0.0684 ; 0.4337] | 0.0533  [0.0161 ; 0.1641] | 0.3484  [0.1650 ; 0.5455] | 0.3736  [0.1686 ; 0.7084] |
| AT | 0.3326  [0.1916 ; 0.4793] | 0.2320  [0.1314 ; 0.3794] | 0.1696  [0.1004 ; 0.2726] | 0.2659  [0.1587 ; 0.3935] |
| CT | 0.3835  [0.1890 ; 0.6338] | 0.1599  [0.0766 ; 0.2694] | 0.0480  [0.0189 ; 0.1290] | 0.4086  [0.1889 ; 0.5891] |
| GT | 0.2763  [0.1057 ; 0.5039] | 0.2568  [0.1287 ; 0.4536] | 0.1256  [0.0559 ; 0.2533] | 0.3412  [0.1627 ; 0.5568] |
| TT | 0.2131  [0.1367 ; 0.3070] | 0.1435  [0.0790 ; 0.2185] | 0.2233  [0.1423 ; 0.3198] | 0.4201  [0.2986 ; 0.5332] |

Estimates (mean and accompanying 95% credibility interval) for the sixteen sets of base frequencies throughout the tree under the context-dependent model with context-dependent model frequencies. The probabilities are grouped by the identity of the immediate preceding site. Note that the model frequencies used in the remainder of the tree are dependent upon the base’s two immediate flanking bases.

**Software information**

The software program in Additional file 5 has been programmed and compiled in Java 1.6 and runs on any system (Windows, Linux, ...) that has at least Java 1.6 installed. The program is not interactive and requires a so-called Java .properties file to run (much like how PAML works). This .properties file specifies the evolutionary model to be used, the required ancestral root distribution, the number of iterations to be performed, ... The software program does not have a graphical user interface and is to be used from the command line as follows (when using 512 Mb of heap space on a linux system from the default directory, i.e. the directory with the SDDA.class file in):
java –Xmx512m –cp colt1.2.0-jdk1.6.0.jar:log4j-1.2.13.jar:. SDDA input.properties

First, the software allows for estimation of evolutionary parameters for 3 different models: the independent general time-reversible model (GTR), the context-dependent GTR model with context-independent model frequencies (GTR16C; see our previous work [1]) and the context-dependent GTR model with context-dependent model frequencies (GTR16CNS; presented in this manuscript). The following files, stored in the default directory when running the software, require additional explanation:

- estimates-0-GTR16CNS.txt to estimates-95-modelname.txt: 6 evolutionary parameters are used per evolutionary context, in the following order of contexts: A-A, A-C, A-G, A-T, C-A, C-C, C-G, C-T, G-A, G-C, G-G, G-T, T-A, T-C, T-G and T-T. In other words, estimates-0-GTR16CNS.txt to estimates-5-GTR16CNS.txt contain the parameters for context A-A, estimates-6-GTR16CNS.txt to estimates-11-GTR16CNS.txt contain the parameters for context A-C, etc. Estimates-0-GTR16CNS.txt corresponds to $q_{A\left|A\to G\left|A\right.\right.}$ in our paper, estimates-1-GTR16CNS.txt to $q_{A\left|A\to C\left|A\right.\right.}$, estimates-2-GTR16CNS.txt to $q_{A\left|A\to T\left|A\right.\right.}$, estimates-3-GTR16CNS.txt to $q_{A\left|C\to G\left|A\right.\right.}$, estimates-4-GTR16CNS.txt to $q_{A\left|G\to T\left|A\right.\right.}$ and estimates-5-GTR16CNS.txt to $q_{A\left|C\to T\left|A\right.\right.}$. The same order applies for the other evolutionary contexts, i.e. estimates-6-GTR16CNS.txt corresponds to $q_{A\left|A\to G\left|C\right.\right.}$, estimates-7-GTR16CNS.txt corresponds to $q_{A\left|A\to C\left|C\right.\right.}$, etc. In the case of the independent GTR model, only estimates-0-GTR1C.txt through estimates-5-GTR1C.txt will be created.
- piA-GTR16CNS-L-iterations-0.txt to piA-GTR16CNS-L-iterations-15.txt (also applies for piC, piG and piU) correspond to the estimates of $\pi_{A\left|A\left|A\right.\right.}$ to $\pi_{T\left|A\left|T\right.\right.}$, in the order described above (i.e. piA-GTR16CNS-L-iterations-1.txt corresponds to the estimates of $\pi_{A\left|A\left|C\right.\right.}$, piA-GTR16CNS-L-iterations-2.txt corresponds to the estimates of $\pi_{A\left|A\left|G\right.\right.}$, etc.).
- root-pi-A-A-A-second-order.txt to root-pi-U-U-U-second-order.txt (example is given here for a second-order Markov chain at the ancestral root sequence) correspond to the ancestral root distribution probabilities $\pi_{AA\left|A\right.}$ (the probability of observing an A at a given site, when that site is preceded by two A’s) to $\pi_{TT\left|T\right.}$ (the probability of observing a T at a given site, when that site is preceded by two T’s).

Second, since many context-dependent models (including the one presented in this paper) assume that the two immediate neighbours of a given site remain unchanged across the length of a branch, we have provided the possibility of splitting up each branch into several pieces of equal length, according to the approach of Hwang and Green [3]. The user can choose, for each branch, the number of desired branch partitions. However, the way to do so is in the current version not very user-friendly as you have to know the names of the internal nodes. We provide an example later on in this supplementary material.

Third, the software allows for calculation of (log) Bayes Factors between 2 given evolutionary models, each with its own specific ancestral root distribution. Bayes Factors are calculated using the model-switch integration scheme of the thermodynamic integration approach [11]. Both annealing and melting integrations can be calculated separately after which the user only needs to calculate the bidirectional mean. For storage reasons, only the likelihood values for both likelihoods as well as the likelihood difference are stored in output files. Storage files associated with the so-called ‘switchmodel’ get an additional ‘-MS’ term in their notation.

We provide the 2 datasets analyzed in this paper in Additional file 5, i.e. AncestralRepeats.inp and Pseudogenes.inp. Each alignment file has the following structure: a first line with only the number of sequences in the file; then, for each organism, its name and sequence separated by whitespace. Allowed character for sequence composition are A, C, G and T/U (all in capitals). We have never tried using complex characters in the species names, so users are cautioned against doing so.

In the .properties file, the fixed underlying tree topology needs to be provided using the Newick format, but without providing branch lengths (in the field: startingtree). Further, the species names cannot be used but are instead replaced by a number: 1 for the first species sequence in the input file, 2 for the second species sequence, etc. Further, an outgroup needs to be provided as well (in the field: outgroup). If the outgroup consists of a single sequence, then simply the number of the sequence will suffice. If the outgroup consists of multiple sequences, then a range of numbers is required, for example: 8-10 for the ancestral repeats dataset.

Further, the .properties file needs to indicate whether or not the alignment contains any gaps by indicating yes/no in the ‘gaps’ field. The length of the alignment needs to be given in the field ‘length’. The root distribution needs to be given (in the rootdistribution field of the .properties file): -1 for an independent root distribution (always the case for independent models), 0 for a zero-order Markov chain at the ancestral root, 1 for a first-order Markov chain at the ancestral root, 2 for a second-order Markov chain at the ancestral root, 3 for a third-order Markov chain at the ancestral root, and finally, 11 for a first-order Markov chain coupled to the context-dependent evolutionary model (for more information, see the manuscript). The number of iterations also needs to be set for Bayes Factor calculations, but is ignored.

When assuming equal rates among sites (i.e. no among-site rate variation), the field ‘rates’ needs to be set to ‘equal’. In the case of among-site rate variation, the field ‘rates’ needs to be set to ‘gamma’ and in the field ‘categories’, the user needs to enter the number of discrete rate categories by which the continuous gamma distribution is approximated (typically, 4 rate categories are used). The field ‘gammaprior’ is usually set to 50, as it contains the upper limit of the uniform prior distribution for the shape parameter. The field ‘discrete’ needs to be set to ‘median’ at all time.

The field ‘method’ in the .properties file indicates whether a regular MCMC run needs to be performed for a given number of iterations (i.e. for estimation of the evolutionary parameters; method = likelihood) or whether a (log) Bayes Factor needs to be calculated (i.e. to compare two evolutionary models with possibly differing root distributions; method = modelswitch). When ‘method’ is set to ‘likelihood’, the field ‘iterations’ is used to determine the number of MCMC iterations to be performed.

When ‘method’ is set to ‘modelswitch’, additional fields are required to perform the Bayes Factor calculation: the field ‘burnin’ sets the number of iterations to be performed as the burn-in sequence; the field ‘Q’ sets the number of iterations to be performed at each step of the thermodynamic integration; the fields ‘knot0’ and ‘knot1’ determine start and end value of the thermodynamic integration, while ‘step0’ indicates the increment/decrement by which to iterate from ‘knot0’ to ‘knot1’. We refer to the thermodynamic integration paper of Lartillot and Philippe for additional explanation on the integration procedure [11]. In the case of a model switch Bayes Factor calculation, settings need to be provided for the second model as well. The same field names are used for this, but need to be preceded by the term ‘switch’, as can be seen from example .properties file Pseudogenes-BF-GTR-GTR16CNS-Annealing.properties.

The following fields must be set as follows and cannot be changed: ancestors = missing; bayesianrates = yes; alpha = estimate; gammaprior = 50; discrete = median; assumptions = no; tree = user-defined; divisions = 0; switchancestors = missing; switchbayesianrates = yes; switchgammaprior = 50; switchdiscrete = median; switchassumptions = no; switchtree = user-defined; switchdivisions = 0.

Additional file 5 contains 4 example .properties files as examples to perform the main functions of the provided software:

- AR-GTR.properties: calculates 100.000 MCMC iterations for the ancestral repeats datasets using the independent general time-reversible model (GTR), without partitioning branches. Note that the GTR model is called GTR1C in the .properties file.
- AR-GTR16CNS-SplitBranches.properties: calculates 100.000 MCMC iterations for the ancestral repeats dataset using the context-dependent model with context-dependent model frequencies (GTR16CNS), using a second-order Markov chain at the ancestral root sequence. This .properties file illustrates how to partition certain branches in 2 or more equal-length pieces.
- Pseudogenes-GTR16CNS.properties: calculates 100.000 MCMC iterations for the pseudogenes dataset using the context-dependent model with context-dependent model frequencies (GTR16CNS), using a first-order Markov chain at the ancestral root sequence.
- Pseudogenes-BF-GTR-GTR16CNS-Annealing.properties: calculates the annealing version of the model-switch integration scheme by performing thermodynamic integration from 0.0 to 0.2 using an increment of 0.001 and performing 1.000 iterations at each step of the way. The models being compared are the independent GTR model and the context-dependent GTR16CNS model (with context-dependent model frequencies) with a first-order Markov chain at the ancestral root sequence. At the end of the calculation, the standard output gives the result of the (log) Bayes Factor calculation, in this case the following is shown on screen:

Quasistatic values: 201

Marginal Likelihood = -311.0956269554793

Combined Marginal Likelihood = -62.219125391095865

Alpha factor: 0.2

Discretization error: 1.6889687211074793

Sampling error: 2.1023265288555244

Confidence interval: [-67.36642125217068 ; -57.07182953002105]

In other words, the log Bayes Factor for the annealing version equals -62.22 log units with a corresponding 95% confidence interval of [-67.37 ; -57.07].
